# Dynamic release of neuronal extracellular vesicles containing miR‐21a‐5p is induced by hypoxia

**DOI:** 10.1002/jev2.12297

**Published:** 2023-01-03

**Authors:** Nea Korvenlaita, Mireia Gómez‐Budia, Flavia Scoyni, Cristiana Pistono, Luca Giudice, Shaila Eamen, Sanna Loppi, Ana Hernández de Sande, Benjamin Huremagic, Maria Bouvy‐Liivrand, Merja Heinäniemi, Minna U. Kaikkonen, Lesley Cheng, Andrew F. Hill, Katja M. Kanninen, Guido W. Jenster, Martin E. van Royen, Laura Ramiro, Joan Montaner, Tereza Batkova, Robert Mikulik, Rosalba Giugno, Jukka Jolkkonen, Paula Korhonen, Tarja Malm

**Affiliations:** ^1^ University of Eastern Finland A.I. Virtanen Institute for Molecular Sciences Kuopio Finland; ^2^ Department of Computer Science University of Verona Verona Veneto Italy; ^3^ Department of Immunology University of Arizona Tucson Arizona USA; ^4^ Department of Human Genetics KU Leuven Leuven Flanders Belgium; ^5^ University of Eastern Finland School of Medicine Kuopio Finland; ^6^ Department of Biochemistry and Chemistry School of Agriculture Biomedicine & Environment La Trobe University Melbourne Victoria Australia; ^7^ La Trobe Institute for Molecular Science La Trobe University Bundoora Victoria Australia; ^8^ Institute for Health and Sport Victoria University Melbourne Victoria Australia; ^9^ Department of Urology Erasmus University Medical Center Rotterdam The Netherlands; ^10^ Department of Pathology Erasmus University Medical Center Rotterdam The Netherlands; ^11^ Neurovascular Research Laboratory Vall d'Hebron Institute of Research (VHIR) Universitat Autònoma de Barcelona Barcelona Spain; ^12^ Institute de Biomedicine of Seville IBiS/Hospital Universitario Virgen del Rocío/CSIC/University of Seville & Department of Neurology Hospital Universitario Virgen Macarena Seville Andalucía Spain; ^13^ BioVendor‐laboratorni medicina a.s. Brno Czech Republic; ^14^ International Clinical Research Center Neurological Department St. Anne's University Hospital and Masaryk University Brno Czech Republic

**Keywords:** biomarkers, extracellular vesicle, hypoxia, ischemic stroke, miR‐21a‐5p, neuron

## Abstract

Hypoxia induces changes in the secretion of extracellular vesicles (EVs) in several non‐neuronal cells and pathological conditions. EVs are packed with biomolecules, such as microRNA(miR)‐21‐5p, which respond to hypoxia. However, the true EV association of miR‐21‐5p, and its functional or biomarker relevance, are inadequately characterised. Neurons are extremely sensitive cells, and it is not known whether the secretion of neuronal EVs and miR‐21‐5p are altered upon hypoxia. Here, we characterised the temporal EV secretion profile and cell viability of neurons under hypoxia. Hypoxia induced a rapid increase of miR‐21a‐5p secretion in the EVs, which preceded the elevation of hypoxia‐induced tissue or cellular miR‐21a‐5p. Prolonged hypoxia induced cell death and the release of morphologically distinct EVs. The EVs protected miR‐21a‐5p from enzymatic degradation but a remarkable fraction of miR‐21a‐5p remained fragile and non‐EV associated. The increase in miR‐21a‐5p secretion may have biomarker potential, as high blood levels of miR‐21‐5p in stroke patients were associated with significant disability at hospital discharge. Our data provides an understanding of the dynamic regulation of EV secretion from neurons under hypoxia and provides a candidate for the prediction of recovery from ischemic stroke.

## Graphical abstract

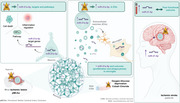

## 1 INTRODUCTION

Hypoxia is a hallmark of several common pathologies such as cancers and myocardial infarction. Thus, understanding the cellular adaptation mechanisms to hypoxia is crucial to efficiently managing these conditions. Hypoxia has been shown to induce the release of extracellular vesicles (EVs) (Bister et al., 2020; Chistiakov et al., 2016; Dong et al., 2021; Guo et al., 2019; Walbrecq et al., 2020) and to have an impact on the composition and function of the EVs (Patton et al., 2020; Tucher et al., 2018; Venturella et al., 2021; Walbrecq et al., 2020; Yaghoubi et al., 2020). Hypoxic EVs enhance the survival and angiogenic capacity of endothelial cells (Bister et al., 2020; Gregorius et al., 2021), have an anti‐inflammatory effect in immune cells (Bister et al., 2020) and enhance tumour progression and invasion by modifying the immunometabolic profile of infiltrating monocytes and macrophages (Park et al., 2019; Zhang et al., 2019). miR‐21‐5p is the most frequently reported hypoxia‐induced microRNA (miRNA) in the EVs (Bister et al., 2020). miR‐21‐5p laden EVs show a positive correlation with metastasis (Roman‐Canal et al., 2019), promotion of angiogenesis in endothelial cells (An et al., 2019) and have been shown to increase migration, expansion and invasion in cancer cells (Guo et al., 2018; Li et al., 2016). In several studies, hypoxia was shown to increase the levels of miR‐21‐5p in the EVs in a hypoxia‐inducible factor‐α (HIFα) dependent manner and these EVs were associated with anti‐inflammatory and anti‐apoptotic effects on the recipient cells (Cui et al., 2018; Pan et al., 2019; Wei et al., 2021). The ability of the EVs to modulate the functions of the recipient cells makes the EVs attractive candidates for therapeutic treatments and the possibility to detect context specific EV cargo alterations from biofluids supports their potential as biomarkers (Karttunen et al., 2019; Xu et al., 2018).

Yet, the existing studies focus on the EVs derived from non‐neuronal cells and it is not known whether neurons respond to hypoxia similarly as described for other cell types. Neurons are highly specialised cells of the nervous system and extremely sensitive to hypoxia due to their high energy demand. The consequences of hypoxia‐induced cell death on the EV secretion from neurons are unknown. Furthermore, the real EV association of miR‐21‐5p, a phenomenon that has been recently questioned in general for all miRNAs (Sork et al., 2021), is currently insufficiently characterised by hypoxic insult.

Here we aimed to fill in the gap in understanding the effects of hypoxia on the neuronal secretion of EVs. We pinpoint miR‐21a‐5p as the most prominently deregulated neuronal miRNA in a mouse model of ischemic stroke and provide data to support the role of miR‐21a‐5p in the regulation of hypoxic responses. Hypoxia induced the secretion of EVs from neurons, and prolonged hypoxia increased the size of neuronal EVs. Finally, our data show that miR‐21a‐5p is secreted via neuronal EVs even prior to the hypoxia‐induced increase at the cellular level, but also in a non‐EV‐associated manner. Furthermore, higher blood levels of miR‐21‐5p at hospital admission are associated with significant disability in stroke patients at hospital discharge.

## 2 MATERIAL AND METHODS

### 2.1 Animals

All experiments using animals had approval from the National Animal Experiment Board of Finland and followed the Council of Europe Legislation and Regulation for Animal Protection. A total of 22, 3–6 months old male Balb/cOlaHsd mice (Harlan Laboratories B.V., Venray, Netherlands) were used for the in vivo studies. Mice were housed under controlled humidity, light, room temperature and food and water were offered ad libitum.

#### 2.1.1 Permanent middle cerebral artery occlusion (pMCAO)

Cerebral ischemia was introduced by occluding the left MCA permanently as described before in detail (Dhungana et al., 2013). Briefly, mice were anaesthetised with 5% isoflurane in a carrier gas mixture of 30% oxygen/70% nitrous oxide and anaesthesia was maintained with 2% isoflurane. The body temperature was monitored and maintained using a heating blanket and rectal probe (Harvard Apparatus, PanLab, Barcelona, Spain). A hole of 1 mm in diameter was drilled in the temporal bone to expose the MCA. The artery was lifted and a thermocoagulator (Aaron Medical Industries Inc., Clearwater, FL, USA) was used for the occlusion. Sham‐operated animals were handled the same way, exposing and lifting the MCA but no occlusion was performed. The skin was sutured after which the animals were placed back in their home cages to recover for up to 5 days.

#### 2.1.2 Perfusion and tissue collection

Mice were anaesthetised with tribromoethanol (250 mg/kg, Avertin®, Sigma‐Aldrich, St. Louis, MO, USA) and perfused brains were collected and divided into ipsilateral (ischemic) and contralateral (healthy) hemispheres. The olfactory bulb and cerebellum were removed. For the RNA analysis of the brain tissue lysates, further, dissection was performed to separate the ischemic lesion core from the surrounding peri‐ischemic cortex. Dissected tissues were rapidly frozen in liquid nitrogen and stored at –70°C prior to analysis.

### 2.2 Cell culture

#### 2.2.1 Primary mouse cortical neurons

Primary cortical neuron (CXN) cultures were prepared from embryonic day 15 C57Bl/6J mouse embryos. Cortices were dissected out and digested with trypsin at 37°C for 15 min, treated with DNase and Soybean trypsin inhibitor (Sigma–Aldrich, St. Louis, MO, USA), washed and counted. The cells were plated on PDL coated cell culture plates in Neurobasal Medium supplemented with B27, 500 μM L‐glutamine and Penicillin/Streptomycin (all from ThermoFisher Scientific, Waltham, MA, USA). On day 5 after culture preparation, half of the media was renewed, and the cells were used for the experiments on day 6 or 7 in culture.

#### 2.2.2 Primary mouse microglia

Primary microglia cultures were prepared from neonatal C57Bl/6J mice using a protocol based on a mild trypsinisation approach as described and characterised before (He et al., 2018; Loppi et al., 2018; Saura et al., 2003). Briefly, the mice were decapitated, brains dissected and mechanically dissociated and incubated with trypsin‐EDTA in microglia base medium containing DMEM‐F12 supplemented with sodium bicarbonate (14 mM), beta‐mercaptoethanol (50 μM), Penicillin‐Streptomycin (100 U/ml and 100 μg/ml, respectively) and HEPES (15 mM), at +37°C for 15 min. The tissue homogenate was transferred into microglia base medium supplemented with 10% heat‐inactivated FBS (complete medium) to inactivate the trypsin and let it settle at the bottom of the tube. Tissue was transferred into fresh complete medium and triturated until completely homogeneous and resulting mixed glial cultures obtained from one neonate brain were plated on 15 cm plates. Cells were cultured for 3 weeks before astrocytes were washed out using trypsin and the remaining microglia cells were collected, washed, and plated for the experiments that were started on the following day. All cell culture reagents were from ThermoFisher Scientific, Waltham, MA, USA.

#### 2.2.3 Immortalised mouse cell lines

Mouse neuroblastoma (N2A), macrophage (RAW 264.7) and endothelial (C166) cell lines were maintained in DMEM, high glucose, GlutaMAX™ Supplemented base medium and microglial (BV2 and N9) cells in RPMI 1640 Medium, GlutaMAX™ Supplemented base medium. The base media were supplemented with Penicillin‐Streptomycin (100 U/ml and 100 μg/ml, respectively) and 10% of heat‐inactivated FBS for continuous maintenance of cultures. All media components were purchased from Thermofisher Scientific Waltham, MA, USA if not otherwise indicated.

### 2.3 Hypoxia and inflammation in vitro models

Lack of oxygen was modelled in vitro either by oxygen‐glucose deprivation (OGD) where the culture medium was depleted from glucose and the cells were incubated in the presence of 1% oxygen or chemically adding cobalt chloride (Sigma–Aldrich, St. Louis, MO, USA) at a final concentration of 300 μM while vehicle control cells received the same volume of sterile water. Normoxic control cells for the OGD were incubated in a normal humidified cell culture incubator. For downstream analysis of the EVs, the hypoxic cultures were carried out without the FBS.

Inflammation was induced in vitro in microglial cell lines by supplementing the culture media with 25 ng/ml of LPS (serotype O55:B5, Sigma–Aldrich, St. Louis, MO, USA).

### 2.4 Extracellular vesicle isolation

To enrich EVs from the cell‐conditioned medium, 35 million CXN cells or 5 million N2A cells were plated on 15 cm culture plates in their normal culture medium. Before transferring the cells to a serum‐free EV collection medium and starting the hypoxia treatments, CXN were left to recover from plating for 6–7 days and N2A for 2 days, during which they reached approximately 80% confluency. Depending on the application, media from 1 to 6 identical plates were pooled together, corresponding to medium volumes between 20 and 120 ml. To inhibit the release of EVs, cells were treated with 5 μM GW4869 (Sigma–Aldrich, St. Louis, MO, USA), or control containing the same concentration of DMSO, for 24 h before the medium was collected for the EV isolation.

After incubation in serum‐free media under desired conditions, the media were immediately separated from the cells and kept on ice, and all the following steps performed at +4°C if not stated otherwise. Serial centrifugation steps of the supernatant were performed to remove any residual cells at 1200 rpm (corresponding to 300 g for this rotor) (Megafuge 1.0R centrifuge, Heraeus, Hanau, Germany) for 5 min and larger debris and vesicles at 12,830 rpm for 20 min using JA‐17 rotor (average g‐force corresponds to 16,500 g) in J2‐MC High Speed centrifuge (Beckman Coulter Life Sciences, Indianapolis, IN, USA). After this step, the pre‐cleared supernatant containing the EVs was further enriched for EVs by ultracentrifugation (UC) or size exclusion chromatography (SEC).

UC at 36,400 rpm for 90 min at +4°C was performed in 26 ml thick wall polycarbonate bottles using Type 50.2 rotor (k‐factor = 131, average g‐force corresponds to 1,20,423 g) in Optima™ LE‐80 ultracentrifuge (all from Beckman Coulter Life Sciences, Indianapolis, IN, USA) to pellet the EVs. The break was set to slow. Samples pooled from several plates were first concentrated to fit into one polycarbonate bottle using Amicon Ultra Centrifugal Filter Units with 10 kDa regenerated cellulose membranes (Sigma–Aldrich, St. Louis, MO, USA). The supernatant was immediately removed completely by 10 ml serological pipette and the bottles were filled with ice‐cold PBS to wash out residual medium contamination by repeating the UC step. The final EV pellet was resuspended in PBS by pipetting up and down 30 times.

Original qEV 70 nm SEC columns (Izon Science, New Zealand) were used as a second EV enrichment method based on the particle size according to manufacturer's instructions. Amicon Ultra Centrifugal Filter Units with 10 kDa regenerated cellulose membranes (Sigma–Aldrich, St. Louis, MO, USA) were used to concentrate the pre‐cleared medium from six pooled 15 cm plates down to 500 μl and the concentrate was stored at –70°C prior further purification. The concentrate was thawed at room temperature and applied to the SEC column. After the void volume, 20 fractions of 500 μl were collected using PBS as an elution buffer. Fractions 1–4 and 13–16 corresponding to the EV and free protein‐enriched fractions, respectively, were pooled together and concentrated down to 150 μl using the Amicon concentrators as earlier.

### 2.5 Western blotting

Proteins were extracted using RIPA buffer and total protein concentrations were quantified by Pierce™ BCA Protein Assay Kit (ThermoFisher Scientific, Waltham, MA, USA). Samples were prepared for gel electrophoresis by boiling in Laemmli sample buffer for 5 min. SDS‐PAGE gels were loaded with 1 μg of total protein from EVs or control cells and tissue lysates. Trans‐Blot Turbo® transfer system (BioRad, Hercules, CA, USA) was used to transfer the proteins on 0.2 μm Immobilon PSQ PVDF Transfer Membrane after which it was blocked with Blok‐CH Reagent for chemiluminescence detection (Sigma–Aldrich, St. Louis, MO, USA). Primary antibodies (Table 1) were diluted in the blocking reagent and incubated overnight at 4°C with gentle agitation. After washing in 0.2% Tween®20 in PBS, HRP‐conjugated secondary antibodies (Table 1) were incubated for 1 h at room temperature before washing and detection with Clarity™ Western ECL Substrate kit and ChemiDoc™ MP Imaging System (both Bio‐Rad, Hercules, CA, USA).

**TABLE 1:** Antibodies

Name	Producer	Catalog #	Method	Dilution
anti‐TSG101, rabbit	Sigma‐Aldrich	T5701	WB	1:2000
anti‐Flotillin‐1, rabbit	Invitrogen	PA5‐17127	WB	1:1000
anti‐syntenin 1 C‐term, rabbit	GeneTex	GTX108470	WB	1:500
anti‐GRP78, rabbit	Invitrogen	PA1‐014A	WB	1:500
anti‐synaptophysin Monoclonal Antibody (SP11), rabbit	Invitrogen	MA5‐14532	WB	1:500
goat anti‐rabbit IgG HRP‐conjugate	Bio‐Rad	170‐6515	WB	1:3000

### 2.6 Nanoparticle tracking analysis

The particle concentration and size distribution were determined using NanoSight NS300 (Malvern Panalytical, Malvern, Worcestershire, UK) using constant settings between the samples. Camera level (brightness of the image achieved with a combination of sutter and gain) was set to 13 for the EVs isolated from cell culture media and four videos of 30 s were acquired for each sample. NanoSight NTA software version 3.2 was used for the analysis of the videos and the detection threshold was kept at 5.

### 2.7 EVQuant

EV quantification was performed using the EVQuant assay as previously described (Hartjes et al., 2020). In short, UC purified EV samples were labeled with the generic fluorescent membrane dye Octadecyl Rhodamine B Chloride (R18) (0.33 ng/μl, 568 nm exc). Samples were mixed with a non‐denaturing polyacrylamide gel solution. The mixtures were transferred to a 96‐well imaging plate (CellCarrier‐96 Ultra Microplates, Perkin Elmer). Immobilised fluorescent EVs were imaged using a confocal Opera Phenix HCS system (Perkin Elmer, Waltham, MA, USA). Individual EVs were detected using the Perkin Elmer Harmony image analysis package v4.9 based on the generic membrane label Octadecyl Rhodamine B Chloride (R18) within a calibrated measurement volume and converted to EV concentration. EV concentration was corrected using a dye only control sample.

### 2.8 Analysis of EV uptake and functions in vitro

To visualise and quantify EV uptake, EVs were labelled using PKH26 generic membrane label (PKH dye, Sigma‐Aldrich, St. Louis, MO, USA) according to the manufacturer protocol. In short, 10^11^ UC isolated EVs (based on the EVQuant measurement using Octadecyl Rhodamine B Chloride (R18)) and 5 μl PKH26‐dye (2.5E‐5 M) were separately diluted in provided Diluent C to 100 μl end volume and subsequently mixed. After 10‐min incubation, unbound PKH26 dye was removed using an Exosome Spin Column (Thermofisher Scientific, Waltham, MA, USA) according to manufacturer's protocol. The PKH26 labeled EVs were quantified by EVQuant to allow equal EV levels in uptake experiments (Hartjes et al., 2020).

To measure EV uptake, 10,000 cells were seeded per well in a 96‐well imaging plate (CellCarrier‐96 Ultra Microplates, PerkinElmer, Waltham, MA, USA) and cultured for 48 h. Cells were incubated with 6 × 10^8^ PKH26 labeled EVs/ml for 0, 1, 2 or 3 h. Cells were fixated with 4% PFA for 30 min. After fixation, the plasma membrane of the cells was stained with PKH67 (2 μl/ml) according to the manufacturer's protocol and nuclei were stained with Hoechst (1:10000).

Fluorescent images were acquired using a confocal Opera Phenix HCS system (PerkinElmer, Waltham, MA, USA) equipped with a 40× water immersion objective. Hoechst, PKH26 and PKH67 were excited using 405, 561 and 488 nm excitation, and detected at 435–480 nm, 570–630 nm and 500–550 nm, respectively. EV uptake was quantified by detecting PKH26 spots in the total cell (PKH67) area and corrected for the number of nuclei in that area (Roobol et al., 2020).

For the analysis of functional responses in the cells, the non‐labeled N2A EVs were quantified using NTA and a total of 5 × 10^9^ to 2 × 10^10^ particles per 96‐well were administered at time 0 and the exposures were repeated after 24 h by changing the fresh media containing the EVs and LPS to induce inflammation. The effects on proliferation (started immediately after the repeated exposure and followed up for 16 h) and phagocytotic capacity (started directly after the proliferation follow‐up) of the exposed cells were observed as described separately.

### 2.9 Cryo‐electron microscopy

Three microliter aliquots of UC purified EV samples were applied onto Quantifoil R 1.2/1.3 holey carbon grids (Quantifoil Micro Tools GmbH, Großlöbichau, Germany), and vitrified by rapid plunging into liquid‐nitrogen cooled ethane in a Leica EMGP vitrification device (Leica Microsystems, Wetzlar, Germany). The vitrified samples were observed in a Talos Arctica transmission electron microscope (Thermo Fisher Scientific, Waltham, MA, USA) operated at 200 kV. The images were recorded with Falcon 3 detector (Thermo Fisher Scientific, Waltham, MA, USA) operated in linear mode, at a nominal magnification of 57,000×, resulting in a final image sampling of 2.6 Å/px. The diameters of the EVs were measured using ImageJ software (National Institutes of Health).

### 2.10 RNase treatment of the EVs

Concentrated EV and protein fractions from SEC isolation were divided into two equal parts immediately after collection. The other half served as a control without enzyme digestion (–) and the other one was digested with Proteinase K and RNase A (+). Briefly, Proteinase K was added at a final concentration of 0.05 μg/μl and incubated at 37°C for 1 h. The residual enzyme was inactivated by quickly heating the sample at 90°C for 5 min, after which it was immediately cooled down on the ice. RNA outside the EVs was then degraded by adding Rnase A at a final concentration of 0.5 μg/μl and incubating at 37°C for 20 min. The sample volume was adjusted to 250 μl with PBS for both (–) and (+) samples and 750 μl of Trizol LS was immediately added and the samples vortexed and stored at –70°C before RNA extraction. All enzymes and reagents were purchased from Thermofisher Scientific Waltham, MA, USA.

### 2.11 RNA extraction

All RNA were extracted using Trizol from cells and tissue samples. Trizol LS was used for the EV samples. All RNA extraction products were from ThermoFisher Scientific Waltham, MA, USA and the procedures were carried out according to manufacturer's instructions.

From cell cultures, RNA was extracted after treatments from the cells plated on 6‐well plates. Culture media were removed, and the cells were washed with PBS and 1 ml of Trizol added per well. Isolated EV samples were adjusted to 250 μl with PBS and 750 μl of Trizol LS was mixed with samples. Samples in Trizol were stored at –70°C before proceeding with the extraction. Frozen brain tissue was placed in 1 ml of Trizol and homogenised immediately and proceeded with RNA extraction.

A synthetic RNA oligo corresponding to cel‐miR‐39‐3p (Integrated DNA Technologies, Coralville, IA, USA) was added to all EV samples prior to adding the chloroform for normalisation control.

### 2.12 Reverse transcription and qPCR

TaqMan™ MicroRNA Reverse Transcription Kit (both ThermoFisher Scientific Waltham, MA, USA) was used to generate cDNA from 40 ng of total RNA for qPCR of mature miRNA according to manufacturer's instructions. StepOnePlus RT‐PCR System (Life Technologies, Carlsbad, CA, USA) was used to perform qPCR using Maxima Probe/ROX qPCR Master Mix. For miRNAs, expression of U6 was used for normalisation except with the EV samples where the synthetic spike‐in control was used. Used primer assays are listed in Table 2.

**TABLE 2:** Taqman qPCR primer assays

Name	Assay ID	Catalog #
cel‐miR‐39	000200	4440887
miR‐21‐5p	000397	4427975
miR‐127‐5p	002229	4427975
U6 snRNA	001973	4427975

### 2.13 Small RNA sequencing

Small RNA libraries were constructed using 20 ng of small RNA which was enriched from total RNA extracted with the miRVana Kit from peri‐ischemic brain tissues collected 3 days after pMCAO induction or sham operation. The Ion Total RNA‐Seq Kit V2 (Life Technologies, Australia) was used to create small RNA libraries which were ligated to adapters containing a unique index barcode (Ion Xpress™ RNA‐Seq Barcode 1–16 Kit, Life Technologies, Australia) according to the manufacturers’ protocol. The yield and size distribution of the small RNA libraries were assessed using the Agilent 2100 Bioanalyzer™ instrument with the High sensitivity DNA chip (Agilent Technologies). Equally pooled libraries were prepared for deep sequencing using the Ion Chef system (Life Technologies) and sequenced on the Ion Torrent S5™ using Ion™ 540 chips (Life Technologies) and 200 bp chemistry (Life Technologies). Pre‐processing of reads, removal of adapters and barcodes were performed using the Torrent Suite (v.5.0.2).

The FASTQ files were quality checked by FastQC and filtered for reads between 20 and 30 bp. The reads were mapped to the mouse genome (mm10) using BOWTIE and GTF files downloaded from miRBase V.21 (Kozomara et al., 2019) to produce counts using HTSeq (Anders et al., 2015). Differentially expressed miRNAs were identified using DESeq2 (Love et al., 2014).

### 2.14 Global run‐on sequencing

Nuclei were extracted from primary mouse cortical neurons treated with 400 μM glutamate or vehicle for 6 h for the global run‐on (GRO) sequencing as described before (Bouvy‐Liivrand et al., 2017). Briefly, after nuclei extraction, the GRO reaction was performed in the presence of labelled UTP (Br‐UTP) at 30°C for 5 min during which RNA polymerases that were transcriptionally active at the time of cell isolation re‐activate and produce nascent RNA. The Br‐UTP‐labelled RNA was immunocaptured using anti‐Br‐UTP beads (Santa Cruz Biotechnology, Inc., Dallas, TX, USA).

The collected RNA was subsequently processed into Illumina sequencing libraries, and single‐end 50 bp sequence reads were generated using Illumina HiSeq2000 as described before (Moreau et al., 2021).

The raw GRO‐seq reads were filtered, aligned to mouse genome mm9 with Bowtie and further processed using the HOMER software suite (Heinz et al., 2010). Differential expression (DE) analysis was performed using the BioConductor limma package (Ritchie et al., 2015) that computes moderate t‐statistics and false discovery rates (Benjamini–Hochberg) to identify significant changes between the glutamate‐treated and untreated neurons. Given the small sample size of the GRO‐seq experiment (n = 2 per condition) and the robust methods for detecting differential expression, loci having nominal *p*‐value of < 0.05 were considered as differentially expressed.

### 2.15 HITS‐clip sequencing

N2A cells were maintained in Kool‐aid High Glucose media (UCSF Cell Culture Facility, Media Production Unit, University of California San Francisco, San Francisco, CA, USA) supplemented with 10% inactivated Fetal Bovine Serum and 1% penicillin‐streptomycin (both ThermoFisher Scientific, Waltham, MA, USA). When the cultures reached 80% confluency, media were replaced with PBS. The cells were crosslinked once at 400 mJ/cm^2^ and after that twice at 200 mJ/cm^2^ by using Stratagene UV Stratalinker 1800 (Stratagene, LaJolla, CA, USA). After crosslinking the cells were scraped off the plates and collected into tubes, pelleted, and stored at –80°C as a dry pellet until further processing. Each sample corresponded to approximately 60 million cells.

The High‐Throughput Sequencing with UV‐crosslinking and Immunoprecipitation (HITS‐clip) procedure was performed as described previously (Moore et al., 2014), with some modifications. Briefly, the frozen samples after crosslinking were lysed and they went through Ago immunoprecipitation and radiolabeling of RNA. Ago‐RNA complexes were further purified by SDS‐PAGE and nitrocellulose transfer and visualised by autoradiography. Products were then extracted from the membrane, amplified with PCR, analysed with Bioanalyzer and sequenced (Illumina).

Genes with a significant peak at the 3′UTR region detected in all the sequenced samples were retrieved from the HITS‐clip sequencing data. The resulting list was used to determine the relative coverage for each *Mus musculus* miRNA by comparison to its total number of 3′UTR targets. The number of peaks falling into the target genes was used to perform an enrichment analysis to test if a miRNA had targets due to random. Finally, the pathways regulated by the miRNA were identified using the Metascape tool (Zhou et al., 2019). Ischemia‐related networks regulated by the miR‐21a‐5p were identified by including genes that closely connect with at least two of the HITS‐clip genes using Ingenuity Pathway Analysis (IPA) software.

### 2.16 MitoSOX and caspase‐3/7 assays

The effects of cobalt chloride exposure on mitochondrial reactive oxygen species (ROS) production and apoptosis were measured in N2A cells using MitoSOX™ Mitochondrial Superoxide Indicator (ThermoFisher Scientific Waltham, MA, USA) and Incucyte® Caspase‐3/7 Green Dye (Essen BioScience Ltd., Hertfordshire, UK), respectively, according to manufacturers’ instructions. The dyes were diluted in serum‐free culture medium with 300 μM of cobalt chloride and added to cells plated on a PDL‐coated 96‐well plate. Images from live cells were acquired immediately and after every hour using IncuCyte® S3 Live Cell Analysis System (Essen BioScience Ltd., Hertfordshire, UK) red (MitoSOX™) and green (Caspase‐3/7) channels. Phase contrast images were used to count the total number of cells and the percentage of the red and green cells.

### 2.17 Proliferation and phagocytosis

Live cell imaging was performed using IncuCyte® S3 Live Cell Analysis System (Essen BioScience Ltd., Hertfordshire, UK) to follow up the proliferation and phagocytosis capacity of EV‐exposed N9 cells.

The phase contrast images were used to calculate the confluency %. Values were turned to fold changes by normalising to the first captured image and plotted against time to estimate the proliferation rate of the cells.

Phagocytosis rate of the cells was determined using pHrodo™ Green Zymosan Bioparticles™ Conjugate for Phagocytosis (ThermoFisher Scientific Waltham, MA, USA) according to manufacturer's instructions. Immediately before adding the reconstituted particles to the cells, a phase contrast image was captured to normalise phagocytosis rate to the cell confluency %. Images were acquired immediately after adding the particles and then every 30 min for a total of 6 h using the green channel. Total Integrated Intensity was quantified and normalised to the cell confluency.

### 2.18 Stroke patients

The study protocol was approved by the Hospital Universitari Vall d'Hebron Ethics Committee [PR (AG) 133‐2015 and PR[AG]157/2011] and all patients or their relatives gave a written informed consent. Forty‐six patients with stroke (20 males, 26 females, 73.7 ± 13.1 years) within 6 h after symptom onset were enrolled at hospital admission in the Emergency Department of Vall d'Hebron University Hospital. Stroke diagnosis was confirmed by neuroimaging, which consisted of CT, CT angiography, and CT perfusion in most cases. At hospital admission, a complete medical history regarding vascular risk factors and medication was obtained. Stroke severity was assessed at 24 h, 48 h and discharge with the NIH Stroke Scale (NIHSS). Outcome was evaluated at discharge and 3 months after stroke with the modified Rankin Scale (mRS). A score 3–6 on the mRS was considered as poor outcome. The second cohort included 23 patients (13 males, 11 females, 64.8 ± 20.4 years) with similar clinical presentations to stroke (e.g., seizures, brain tumors, sepsis, migraine and headache). The third cohort included 10 healthy control patients (seven males, three females, 74.2 ± 0.8 years).

Peripheral blood samples were collected in tempus tubes at admission (<6 h after symptoms onset, 3.5 h mean time) before any treatment was given. RNA was isolated using Maxwell® 16 Total RNA Purification Kit (Promega, Madison, WI, USA). RNA concentration and quality was explored with NanoDrop Microvolume Spectrophotometer. RNA isolates were stored at –80°C until use. RNA isolates were analysed using miREIA kit hsa‐miR‐21‐5p (BioVendor‐laboratorní medicína, Brno, Czech Republic) according to manufacturer's protocol. In short, a total of 20 μl of diluted RNA isolates, standards (serially diluted from 12.5 amol/μl to 0.39 amol/μl) or quality controls were mixed with 20 μl of specific DNA oligonucleotide probe and hybridised by a program which started at 85°C (3 min), followed by 4°C (2 min) and finished at 37°C (5 min). Hybrids prepared in the previous step were 3‐times diluted in dilution buffer and pipetted into wells on miREIA microtiter plate. After incubation (1 h) at 37°C plate was washed, and Streptavidin‐HRP conjugate was added into each well. Another incubation at 37°C (30 min) was followed by a second washing and then the addition of substrate solution into each well. The plate was covered by dark foil to avoid light and incubated 15 min at room temperature. Stop solution was added after 15 min as the final step in the assay and absorbance was measured at 450 nm corrected for the absorbance at the reference wavelength 630 nm.

### 2.19 Figures and data analysis

Scientific illustrations were created with BioRender.com. Data figures and graphs were created using following Python packages: pandas, matplotlib.pyplot, seaborn and pingouin. Data are presented as mean ± standard deviation (SD). Statistical analysis was performed using statistical package Pingouin v0.3.11 written in Python 3.

Data distribution was tested for equal variances using Levene's test and normal distribution with Shapiro‐Wilk test. Comparison of groups with equal variances was done using parametric tests like Student's T test or One‐way ANOVA with Tukey's post‐hoc test, where appropriate. Pairwise T tests were used for dependent samples. Mann‐Whitney U test was chosen for non‐parametric option for comparison of two groups. *p*‐values < 0.05 were considered to be statistically significant and the level of significance is indicated in the figures as follows: ***p < 0.001, **p < 0.01, *p < 0.05.

## 3 RESULTS

### 3.1 The levels of miR‐21a‐5p are increased in the in vitro and in vivo stroke models

**FIGURE 1:**
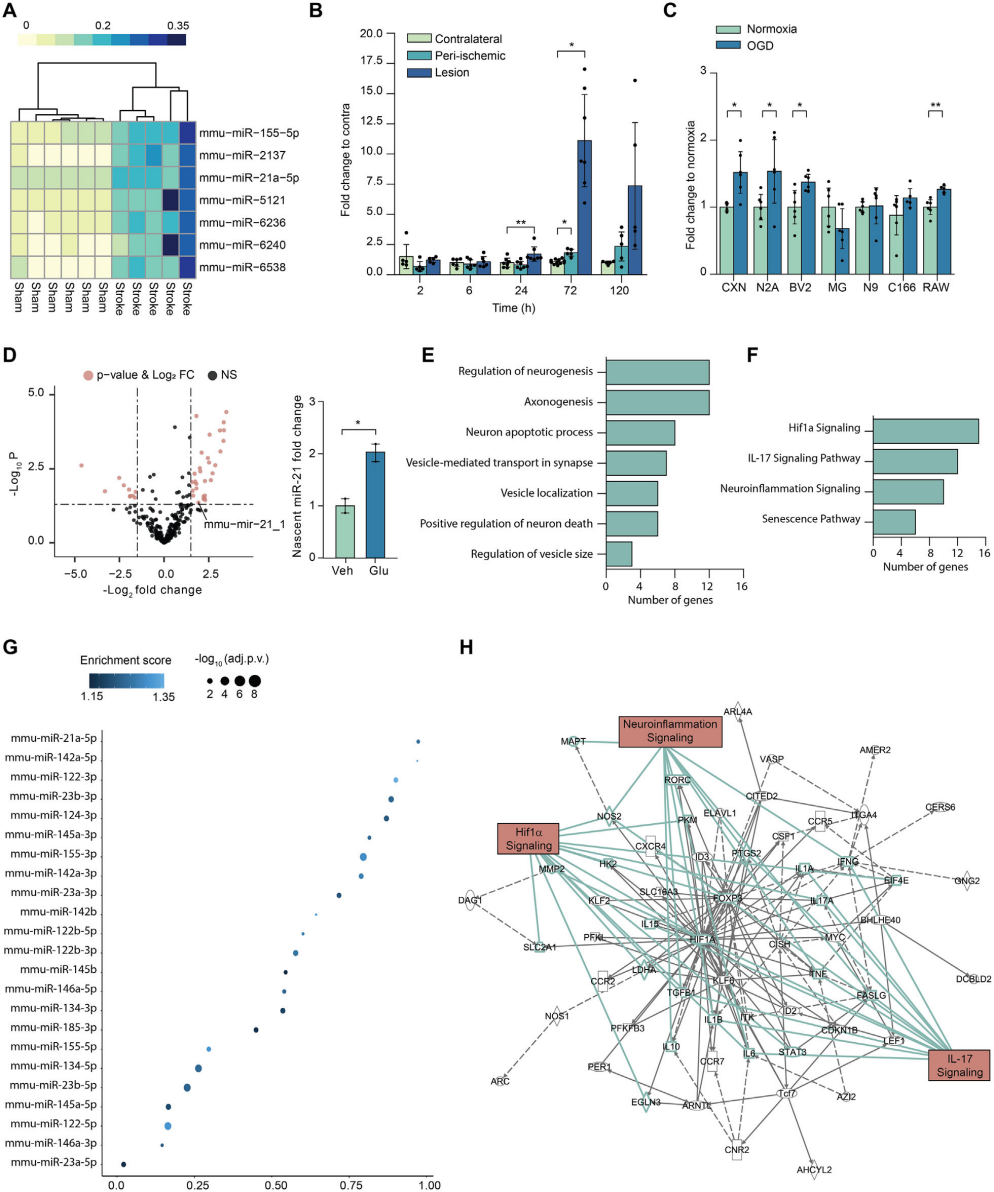
**Ischemic stroke alters the levels of miR‐21a‐5p in the brain and oxygen‐glucose deprived neurons. A**. Heatmap of differentially expressed miRNAs from small RNA sequencing of the mouse brain tissue 3 days post‐permanent middle cerebral artery occlusion (pMCAO). **B**. Validation of miR‐21a‐5p alteration in the mouse brain tissue up to 5 days post‐pMCAO by qPCR (n = 5–6). One‐way ANOVA and Tukey's post hoc for each timepoint was performed. **C**. The regulation of miR‐21a‐5p levels were additionally studied in different primary and immortalised mouse cell lines in normoxia and oxygen‐glucose deprivation (OGD) at the 12‐h timepoint. The dots correspond to technical replicates (n = 4–6) and T‐test between treatment and control of each cell line was performed. **D**. Volcano plot showing changes in the transcriptional activity of miRNA genes in mouse primary cortical neurons after 6‐h glutamate exposure as quantified from GRO‐seq. Bar plot shows the quantification of fold change for miR‐21 (n = 2). BioConductor limma package (Ritchie et al., 2015) that computes moderate t‐statistics and false discovery rates (Benjamini‐Hochberg) was performed. **E**. Pathway enrichment analysis of HITS‐clip miR‐21a‐5p target genes using Metascape. **F,H**. Network construction of enriched pathways using IPA. The list of miR‐21a‐5p bound putative targets in N2A cells were obtained from HITS‐clip analysis and extended with functionally connected genes using IPA software. **G**. Relative coverage and miRNA enrichment of miRNAs of interest obtained from HITS‐clip analysis. B, C and D data are represented as mean ± SD and the significance is stated as ***p < 0.001, **p < 0.01, *p < 0.05

miRNAs have key roles in the regulation of cellular responses to hypoxia. To discover regulators of cellular responses to ischemic conditions in vivo, we carried out miRNA sequencing of mouse brain tissue 3 days after permanent middle cerebral artery occlusion (pMCAO) (Figure 1A). Among significantly altered miRNAs, miR‐21a‐5p showed the strongest differential expression. The expression level of this miRNA is well conserved across species, and thus, we selected this miRNA for further validation. Ischemic core showed increased miR‐21a‐5p levels 24 h post‐pMCAO, peaking at 3 days (Figure 1B). In the peri‐ischemic cortex, a region surrounding the ischemic core, a modest but significant increase of miR‐21a‐5p was detected at 3 days post‐pMCAO.

The role of miR‐21a‐5p has been studied in multiple cell types but data regarding its enrichment in different central nervous system (CNS) cell types remains inconclusive (Buller et al., 2010; Jovicic et al., 2013). To investigate this and to further connect the level of miR‐21a‐5p to stroke related stress, we investigated the miR‐21a‐5p regulation in vitro in several primary and secondary cell lines exposed to OGD. We observed a consistent upregulation of miR‐21a‐5p in primary mouse cortical neurons and in neuroblastoma cell line (N2A) following 12 h of OGD (Figure 1C). Interestingly, this increase was not detected in primary mouse microglia (MG) and data from microglial (BV2 and N9) and macrophage (RAW) cell lines were inconsistent (Figure 1C). Similarly, the endothelial cell line (C166) failed to upregulate miR‐21a‐5p following OGD, suggesting that the hypoxia‐induced miR‐21a‐5p expression is more prominent in neurons. To further evaluate stroke‐induced transcriptional changes of miR‐21 beyond OGD, we carried out GRO‐sequencing of mouse cortical neurons exposed to the excitatory amino acid glutamate mimicking stroke‐related glutamate excitotoxicity and detected glutamate‐induced acute transcriptional changes of several miRNAs (Figure 1D). In this dataset, 108 genes were found differentially expressed with adjusted *p*‐value below 0.05 and 1504 genes with a nominal *p*‐value below 0.05. From 470 pri‐miRNA loci annotated by intersecting mature miRNA genomic coordinates with primary transcripts, 315 (67%) were found to be expressed in the neurons. A total of nine pri‐miRNA loci were differentially expressed with an adjusted *p*‐value below 0.05 and 56 with a nominal *p*‐value below 0.05. Indeed, we observed a trend of miR‐21 TSS1, annotated as mir‐21a, transcriptional upregulation at 6 h post‐exposure to glutamate (nominal *p* = 0.01735, adjusted *p* = 0.2221, Figure 1D).

To predict the consequences of miR‐21a‐5p deregulation in the neuronal cells and its relevance for the ischemic response, we carried out HITS‐clip sequencing of N2A cells under basal conditions. The complete list of all identified miRNAs and their targets, as well as target gene‐enriched pathways for selected miRNAs, are presented in Supplementary File 1. We used the relative HITS‐clip target coverage, compared to the total miRNA targets, and the enrichment score of miRNAs to select miRNAs of interest (Figure 1G). The analysis showed that miR‐21a‐5p is one of the most covered miRNAs in N2A cells. We retrieved 186 putative miR‐21a‐5p target genes and as expected, the enriched pathways included processes related to the neuronal functions and regulation of cell death (Figure 1E). In addition, we discovered a few enriched terms related to the regulation of neuronal vesicles. To discover ischemia related processes which are likely regulated by the miR‐21a‐5p, we extended the HITS‐clip target gene list with tightly functionally connected genes and performed pathway analysis with the resulting gene lists. The pathway analysis indicated gene enrichment in several processes that are important for cellular adaptation to ischemic stroke, including cell death and regulation of inflammation (Figure 1F). As expected, the network construction analysis indicated a tight connection to the HIF1α signaling pathway, a central pathway induced in response to hypoxia (Figure 1H).

Since the effects of hypoxia on the release of extracellular vesicles have been studied in several contexts earlier (Bister et al., 2020), but the studies in the central nervous system and neuronal cells are still lacking, we chose to study this phenomenon in more detail.

### 3.2 Extracellular vesicles secreted by neuronal cells are taken up by several recipient cells

**FIGURE 2:**
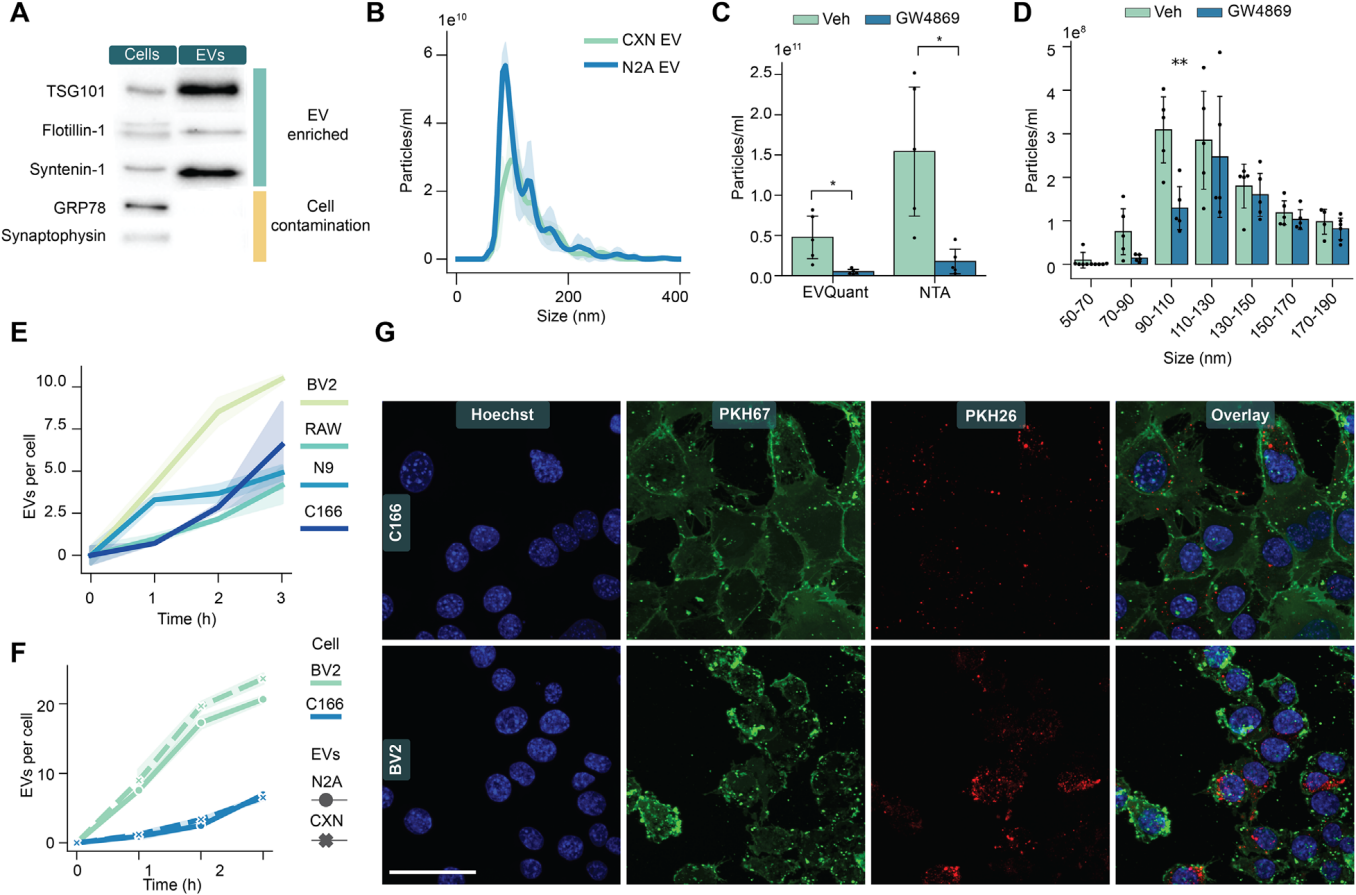
**Extracellular vesicles from neuronal cells are robustly taken up by phagocytic cells. A**. Western blot of the EV enriched markers (TSG‐101, Flotillin‐1, Syntenin‐1) and intracellular contamination markers (GRP78, synaptophysin) in cortical neuron derived EVs and cell lysates. **B**. Size distribution of the cortical neuron and N2A cell derived EVs by NTA (n = 3). **C**. Particle concentrations of the EVs from N2A cells after GW4869 exposure quantified by NTA and EVQuant. Mann‐Whitney U test for unequal variances was performed. **D**. Particle concentrations based on specific sized particles upon GW4869 exposure measured by NTA. Comparison between control and treatment was performed with T‐test except for the 70–90 nm bin where Mann–Whitney U test for unequal variances was used. **E**. Uptake of PKH26 stained cortical neuron EVs by different cell lines quantified by confocal microscopy (n = 3). **F**. Comparison of the uptake kinetics of CXN and N2A cell EVs in microglial and endothelial cell lines (n = 3). Statistical significance for both EV types was found with Two‐way ANOVA test, *p* < 0.001 for both time and cell type. **G**. Representative images of the EV uptake in C166 (upper panel) and BV2 (lower panel) cells at 3 h. Green = lipid membranes of the cells stained with PKH67, blue = nuclei stained with Hoechst, red = EVs stained with PKH26. Scale bar = 50 μm. The dots in C and D correspond to technical replicates (n = 4–6). The data is represented as mean ± SD and the significance is stated as ***p < 0.001, **p < 0.01, *p < 0.05

To evaluate if hypoxia induced miR‐21a‐5p deregulation is associated with altered EV release from neuronal cells, we isolated EVs from mouse cortical neurons and N2A cells using differential ultracentrifugation. The isolated EVs were characterised by Western blotting for the presence of EV associated proteins and shown to be enriched in TSG‐101 and syntenin‐1 when compared to the total cell lysates (Figure 2A). The absence of detectable endoplasmic reticulum protein, GRP78, and synaptic vesicle protein, synaptophysin, suggest that the EV preparation was free from intracellular contamination.

EVs isolated at basal conditions from serum‐free medium of mouse cortical neurons and N2A cells showed no significant difference in size distributions (Figure 2B). To further support the successful isolation of the EVs, N2A cells were treated with GW4869, an inhibitor of nSMase2, to block the release of EVs that depend on ceramide synthesis. This process has been previously shown to have a key role in the EV release from the neuronal cells (Guo et al., 2015). Indeed, we detected a significant decrease in the total EV concentration as measured by two distinct technologies (Figure 2C) while this treatment did not affect the viability or proliferation of the cells (Figure S1). Furthermore, the NTA measurement suggests a specific reduction of the release of EVs between 90 and 110 nm in diameter (Figure 2D).

To transfer messages between cells, the EVs need to interact with the target cells. We exposed several cell lines to PKH26 stained cortical neuron EVs and followed the uptake of the EVs using confocal microscopy (Figure 2E). Interestingly, the EV uptake in microglia (BV‐2 and N9) and macrophage (RAW) cell lines was higher compared to the endothelial cell line (C166). When the cells were exposed to equal numbers of EVs derived from N2A and cortical neurons, we found highly similar uptake kinetics of both type of EVs in microglia but to significantly less extend in endothelial cell lines (Figure 2F,G), suggesting interaction preference of both neuronal model EVs with microglial cells.

### 3.3 Hypoxia induces the release of neuronal EVs and EV associated miR‐21a‐5p before the cellular level of miR‐21a‐5p increases

**FIGURE 3:**
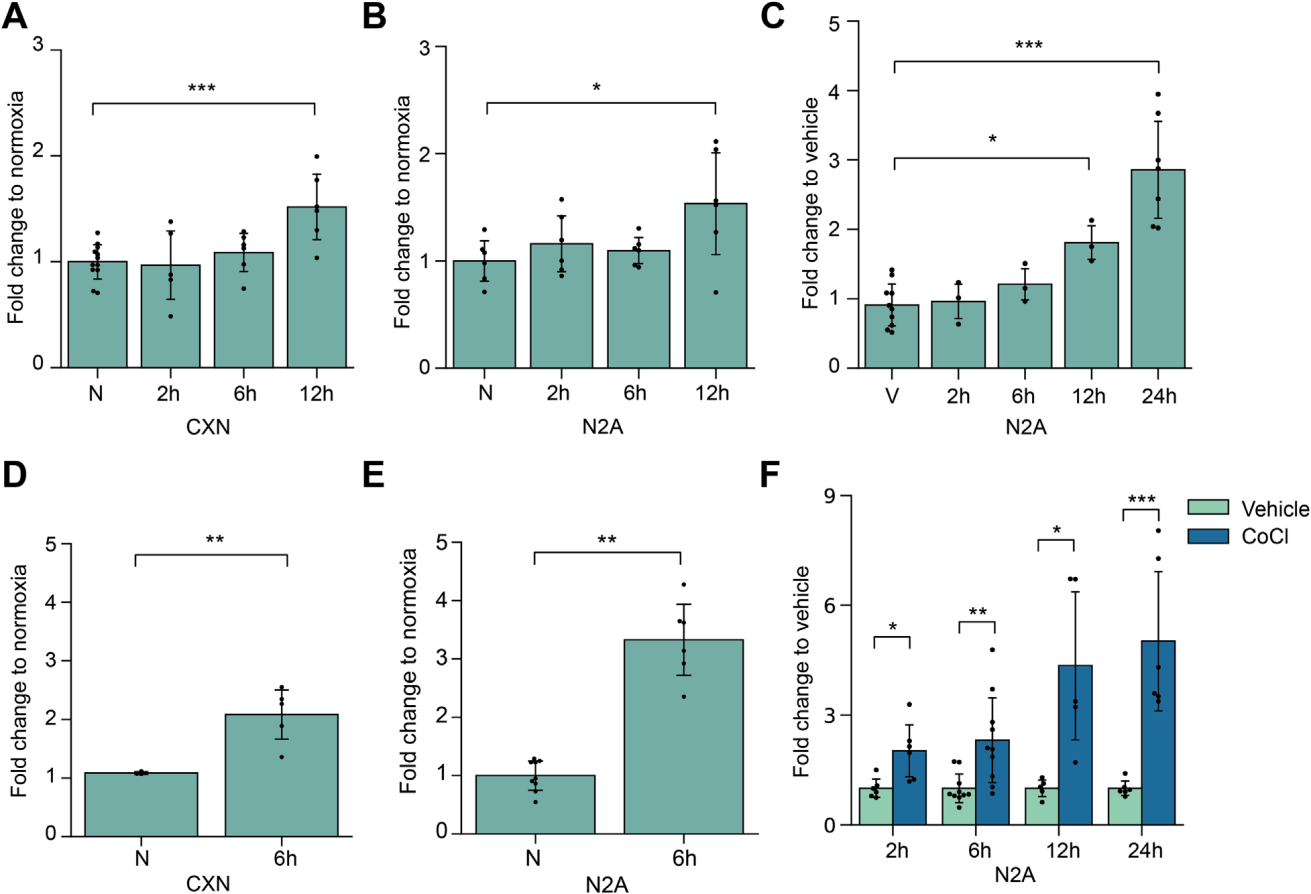
**EV miR‐21a‐5p increase precedes changes at the cellular level under hypoxic conditions. A,B**. Cellular levels of miR‐21a‐5p measured after different lengths of OGD by qPCR from the cortical neuron and N2A cells, respectively. One‐way ANOVA and Tukey's post hoc tests were performed. **C**. Cellular levels of miR‐21a‐5p measured after different lengths of cobalt chloride exposure from N2A cells. One‐way ANOVA and Tukey's post hoc tests were performed. **D,E**. EV miR‐21a‐5p levels measured 6 h after OGD by qPCR from the cortical neuron and N2A cell EVs, respectively. For D, T‐test was performed and for E, Mann‐Whitney U test for unequal variances was performed. **F**. EV miR‐21a‐5p levels measured after different lengths of cobalt chloride exposure from N2A cell EVs. The dots correspond to technical replicates (n = 5‐10). Differences between control and treatment were found with T‐test, except for the 6‐h timepoint where unequal variances were detected, and Mann–Whitney U test was performed. The data is represented as mean ± SD and the significance is stated as ***p < 0.001, **p < 0.01, *p < 0.05

To evaluate the time‐dependent regulation of miR‐21a‐5p at the cellular and EV level under hypoxic conditions, we exposed primary cortical neurons and N2A cells to different lengths of OGD or cobalt chloride treatment and followed the miR‐21a‐5p level by qPCR. We observed similar, statistically significant cellular upregulation of miR‐21a‐5p in both cell types, starting at 12 h of OGD (Figure 3A,B). Another type of hypoxia, induced chemically using cobalt chloride, showed similar dynamic regulation of miR‐21a‐5p in the N2A cells (Figure 3C). Interestingly, under these conditions, an increase of miR‐21a‐5p in secreted EVs could be observed well before the increase at the cellular level, starting as early as 2 h after the exposure (Figure 3D‐F).

The secretion of miR‐21a‐5p into EVs followed a time‐dependent increase in cobalt chloride exposed samples compared to vehicle treated controls (Figure 3F). As cell death can compromise the purity of the isolated EVs, and the generally accepted guidelines highlight the importance of high cell viability for the EV studies (Théry et al., 2018; Witwer et al., 2013), we next evaluated our experimental setup and resulting EV preparations in more detail.

### 3.4 EV preparations from dying neurons differ in abundance, size and contamination profile compared to the healthy cell isolated EVs

**FIGURE 4:**
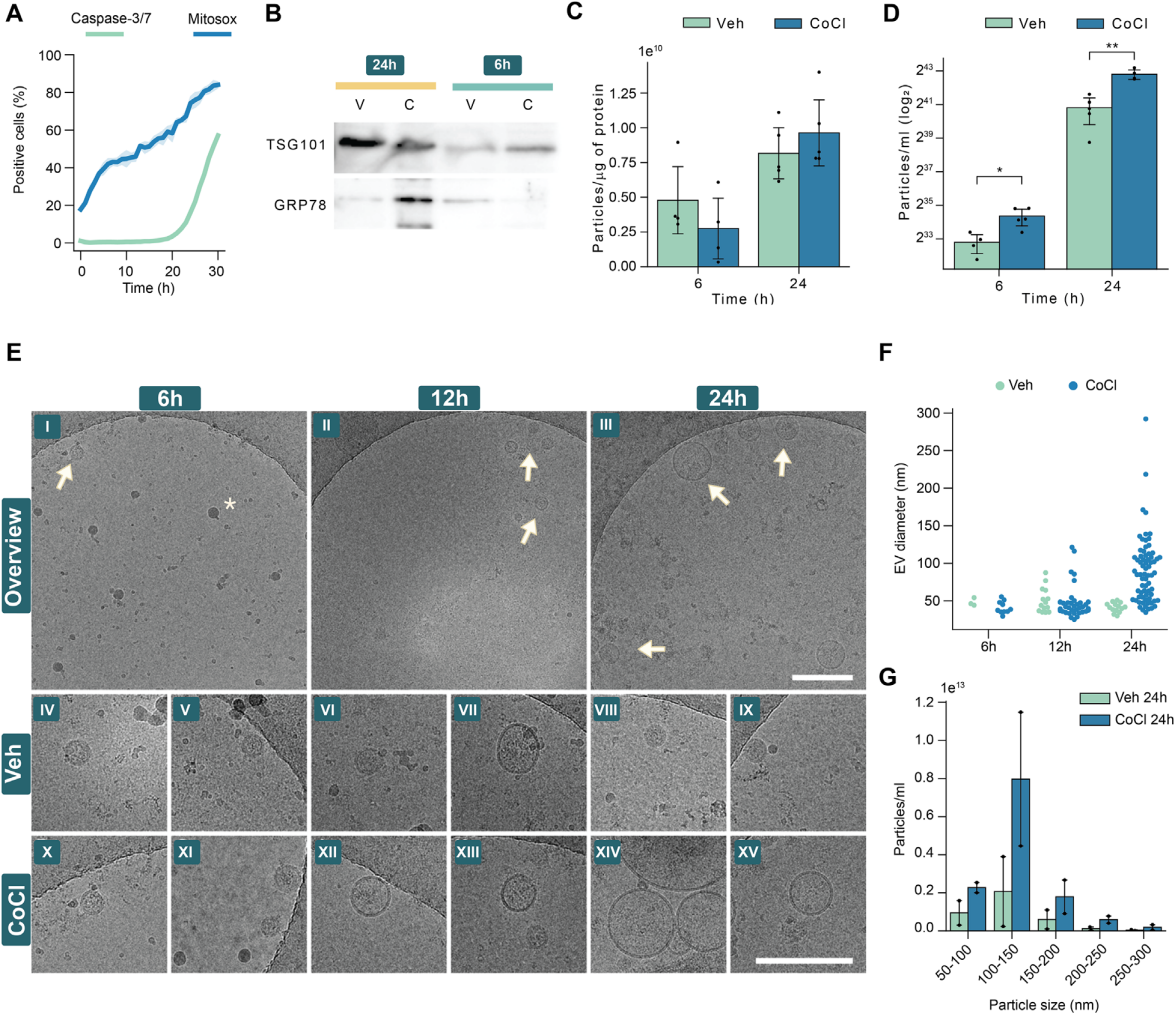
**Prolonged hypoxia can affect the viability of the neuronal cells, compromise the purity of isolated EVs, and alter the size and abundance of the EVs**. **A**. Percentage of MitoSOX or caspase‐3/7 positive cells over time after cobalt chloride exposure. N2A cells were exposed to 300 μM cobalt chloride in serum‐free medium at time point 0 and the fluorescence signal from the assay probes was acquired with IncuCyte® S3 Live Cell Analysis System. **B**. Western blot analysis of isolated N2A cell EVs for EV enriched protein TSG‐101 and intracellular contaminant GRP78 after different cobalt chloride exposure times. **C**. Ratio of particles and total protein after 6‐ and 24‐h timepoints. **D**. NTA measurement of isolated EVs after cobalt chloride exposure of N2A cells. **E**. Cryo‐EM images of isolated EVs. Upper panel (I‐III) shows representative images of the cryo‐EM grids of the EVs isolated after different lengths of cobalt chloride treatment. Lower panel (IV‐XV) shows close ups of the EVs from all timepoints and comparison of the EVs between cobalt chloride and vehicle treatment at each timepoint. Scale bars = 200 nm. **F**. Measured diameter of each observed lipid bilayered vesicle in cryo‐EM images. For 24 h cobalt chloride group, only part of the detected EVs were measured. **G**. Concentrations of different sized particles measured by NTA from isolated EVs. The dots in C, D and G correspond to technical replicates (n = 2‐5). For C and D, a T‐test was used to detect the significant differences. The data is represented as mean ± SD and the significance is stated as ***p < 0.001, **p < 0.01, *p < 0.05

We selected cobalt chloride to induce hypoxia due to its superior technical feasibility and reproducibility. To evaluate the cellular responses to the cobalt chloride treatment, we measured the mitochondrial ROS production as a sign of celullar stress and the rate of apoptosis over the time course used for the EV isolation. Expectedly, a gradually increasing mitochondrial ROS production was observed after the exposure to cobalt chloride while the proportion of apoptotic cells started to rapidly increase after the first 20 h of incubation (Figure 4A). To further analyze the possible consequences of this reduced viability on the EV sample purity, we evaluated the presence of non‐EV proteins and EV enriched proteins at these timepoints in the ultracentrifugation isolated EV preparations (Figure 4B). Interestingly, the detection of GRP78, an endoplasmic reticulum protein, generally considered as a contaminant of EV preparations from the cell cultures (Théry et al., 2018), suggest possibly increased intracellular contamination after 24 h’ cobalt chloride treatment compared to the vehicle.

The ratio of particles and total protein has been suggested as a one type of measure for the EV purity (Webber & Clayton, 2013) as the EVs tend to contain relatively low levels of protein. We measured the number of particles using the NTA in EV preparations isolated after 6‐ or 24‐h cobalt chloride or vehicle exposure and calculated the number of EVs released per microgram of total protein (Figure 4C). No statistically significant differences between these ratios were detected between any of the study groups, suggesting that this type of purity measure may not be suitable for detection of contamination from all sources. Interestingly, cobalt chloride induced a statistically significant increase in the release of particles at both time‐points, with more robust induction at the later timepoint (Figure 4D). It is worth mentioning that for the EV miR‐21a‐5p data (Figure 3D‐F) we used synthetic spike‐in control miRNA for the normalisation as described in Section 2. Thus, we did not account for the increased number of the EVs in the cobalt chloride treatment and the increase of EV miR‐21a‐5p could be partly explained by the increased number of EVs under hypoxia. However, we did not have statistically significant increase of another miRNA, miR‐127, in the same EV samples (Figure S2).

Finally, we carried out cryo‐EM imaging to visualise whether hypoxia alters the EV morphology (Figure 4E). At 6 and 12 h, very few lipid bilayered EVs were present. However, after prolonged cobalt chloride treatment for 24 h, a clear increase in the abundance of double membrane vesicles can be visually observed. To illustrate the apparent difference between the vesicles of 24‐h cobalt chloride treatment, compared to any other group, we measured the diameters of all observed lipid bilayered vesicles (Figure 4F). As evident from the illustration, we were able to detect only three EVs among the images from 6 h vehicle group. This number of detected EVs gradually increase over time. Interestingly, at each timepoint, we were able to detect higher number of vesicles in the cobalt chloride group compared to the vehicle control. At 24‐h cobalt chloride samples, the lipid bilayered vesicles had larger diameter but the diameter remained in the range of what is considered to be the size of small EVs (mean diameters were 41 and 90 nm for vehicle and cobalt chloride 24 h samples, respectively). We tested if this apparent difference in the abundance of larger EVs observed in the cryo‐EM can be recapitulated with the NTA which is currently much more frequently used technique in the EV studies. However, the clear difference seen in the cryo‐EM images was not detected by the NTA (Figure 4G), highlighting the limitations of this technique in size determination of heterogeneous samples with a practical example.

In summary, the use of several methodologies is necessary to adequately understand the effects of different conditions on EV secretion and, especially, how its affected by the cell death.

### 3.5 Most of the miR‐21a‐5p in the neuron conditioned medium is non‐EV associated but the EV associated fraction is well protected from enzymatic degradation

The concomitant observation of intracellular contaminants in the EV preparations, and the reduced viability of the cells at the 24 h cobalt chloride timepoint, made us question the identity and biogenetic origin of these abundant, larger EVs and the association of miR‐21a‐5p within these EVs. As it is still debated, even in the context of healthy cells, if miRNAs are carried inside of the EVs, or rather bound on the surfaces or present in the enriched EV samples as impurities, we aimed to study this concept in more detail using another method to purify EVs from other cell culture medium components. We selected size exclusion chromatography (SEC) to purify EVs based on their size as it could yield distinct non‐EV contaminant profiles compared to the density‐based ultracentrifugation. In addition, we took advantage of the possibility to analyse both the EV enriched fractions and the non‐EV associated, protein enriched, fractions that become easily accessible with the SEC.

**FIGURE 5:**
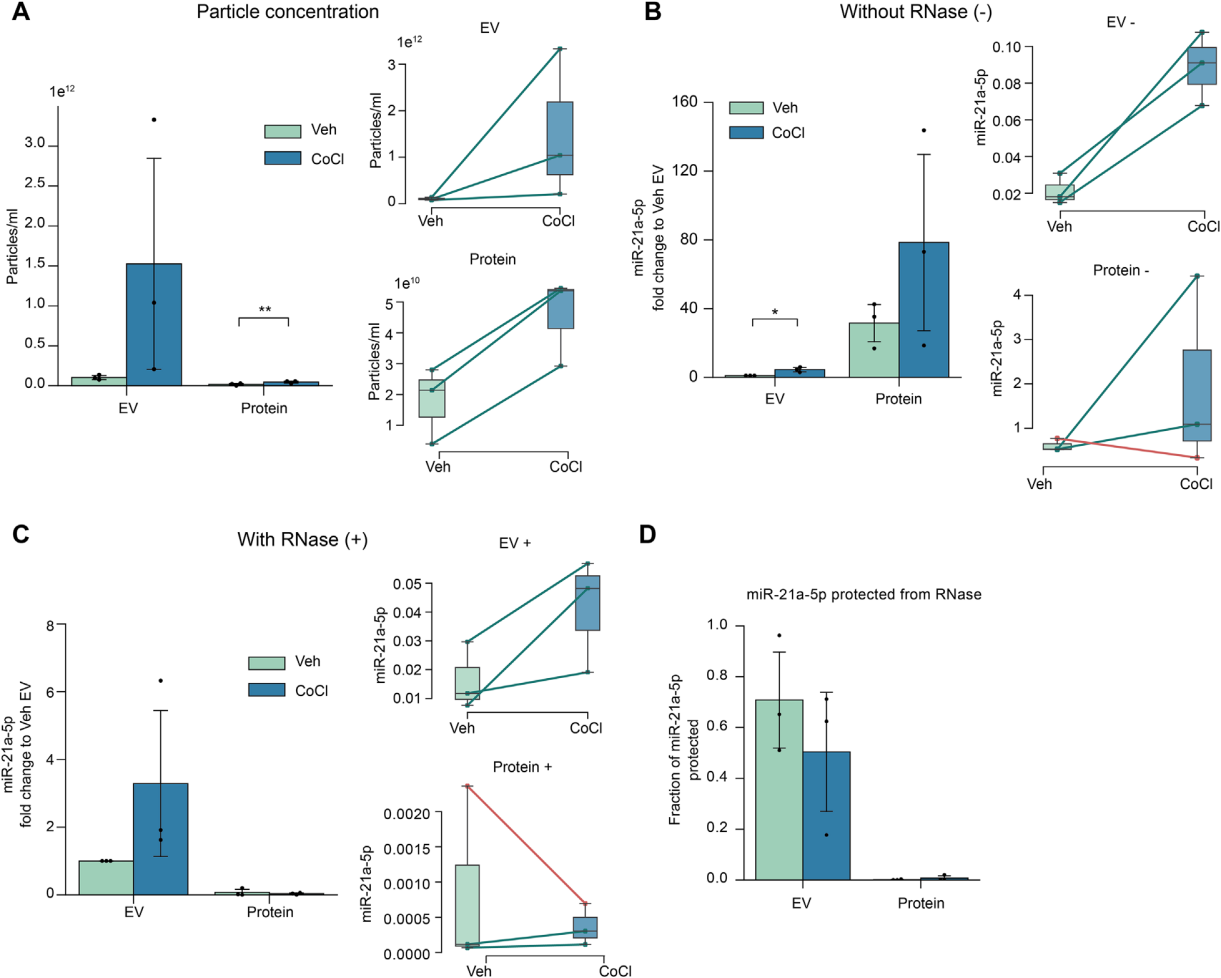
**miR‐21a‐5p is released from neuronal cells in the EVs but also as a non‐EV associated form. A**. Particle concentrations of the EV (SEC fractions 1–4 pooled) and protein fractions (SEC fractions 13–16, corresponding to the peak eluting protein, pooled) from 24 h vehicle or cobalt chloride treated N2A cells measured by NTA. **B**. Relative miR‐21a‐5p levels in the EV and protein fractions measured by qPCR without RNase treatment (–). **C**. Relative miR‐21a‐5p levels in the EV and protein fractions after RNase treatment (+) measured by qPCR. **D**. Fraction of total miR‐21a‐5p protected from subsequent RNase treatment. The inserts in the figures illustrate the measurements separately for each three independent trials (technical replicates n = 5). Statistical significance was calculated using pairwise T‐test between vehicle and cobalt chloride treatments. The data is represented as mean ± SD and the significance is stated as ***p < 0.001, **p < 0.01, *p < 0.05

The majority of the EVs, being larger in size compared to the soluble material, elute before SEC fraction 5, well before the peak of the non‐EV associated proteins that can be detected in the later fractions (Figure S3). To study the distinct characteristics of the EV and protein fractions, we pooled together SEC fractions 1–4 and 13–16 to analyse EVs and proteins separately from the N2A conditioned medium, respectively. The 24 h cobalt chloride exposure yielded higher particle concentrations compared to the vehicle treatment in the protein fraction (Figure 5A), indicating that the fractions with high protein concentration, and practically no EVs present, contain non‐EV particles detected by the NTA, which are affected by the exposure. In the EV fractions we could observe similarly higher particle concentrations after the cobalt chloride treatment compared to vehicle, but this difference did not reach statistical significance (Figure 5A). However, the absolute particle concentrations were approximately 6 and 33 times higher in the EV fractions compared to the protein fractions for vehicle and cobalt chloride groups, respectively. While the particle concentration of protein fractions was very low in comparison to the EV fractions, these data highlight the known bias of NTA technology to detect also non‐EV particles and, based on the size distribution plots, one could falsely argue having EV‐enriched sample (Figure S3).

The relative levels of miR‐21a‐5p were found to be 20‐ to over 100‐fold higher in the protein fractions compared to the EV fractions from vehicle treated cells (Figure 5B). However, we observed higher miR‐21a‐5p level in the EVs from the cobalt chloride treated cells compared to the vehicle treatment while this difference did not reach statistical significance in the protein fractions. To further analyse the possibility, that 24 h cobalt chloride treatment induced cell death could hamper the purity of the isolated EVs and explain the increased miR‐21a‐5p in the EV fractions, we tested for the stability of miR‐21a‐5p upon RNase treatment of SEC fractions. It has been shown that the lipid membrane of the EVs can protect RNA species inside the EVs from the RNase activity and this has been used before to support the miRNA localisation inside the EVs (Cheng et al., 2020). We separated EV and protein fractions with the SEC and divided the samples in half for the enzymatic digestion or no digestion and measured the resulting miR‐21a‐5p levels. After RNase treatment, we failed to reach a statistically significant difference between EV fractions from vehicle and cobalt chloride treated cells although a similar trend of higher miR‐21a‐5p after cobalt chloride treatment remained in the EV fractions (Figure 5C), as observed without RNase treatment. Drastic difference in the stability of miR‐21a‐5p in the EV fractions compared to the protein fractions, where less than 1 % of the total miR‐21a‐5p was protected from enzymatic degradation, was observed (Figure 5D). However, it is worth mentioning that the protected miR‐21a‐5p fraction of the EVs from the cobalt chloride group was lower than in the vehicle group but did not reach statistical significance due to highly variable protection.

Taken together, our data suggest that a considerably large part of miR‐21a‐5p in the neuronal cell culture medium is non‐EV associated but the small fraction incorporated into the EVs is well protected from the enzymatic degradation.

### 3.6 EVs from hypoxic neurons do not affect microglial proliferation or phagocytosis

**FIGURE 6:**
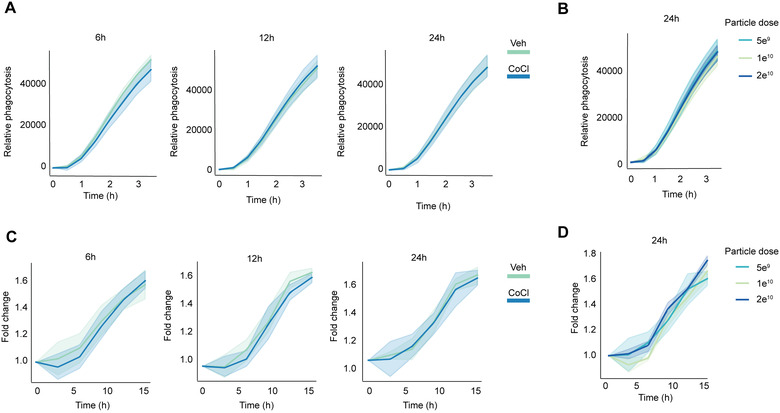
**Microglial cells exposed to hypoxic or normoxic neuronal EVs show no differences in phagocytosis or proliferation rates**. Phagocytosis (A) and proliferation (C) of N9 cells co‐exposed to N2A cell EVs from 6–24 h cobalt chloride treatment and LPS. Phagocytosis (B) and proliferation (D) dose‐response of N9 cells to different cobalt chloride EV doses without concomitant pro‐inflammatory stimulus. Proliferation time was measured after two doses of EVs (once every 24 h). Co‐exposure with LPS for the last 24 h period was applied in (A) and (C). Figures show representative results from one experiment (n = 4 technical replicates). The data is represented as mean ± SD and the significance is stated as ***p < 0.001, **p < 0.01, *p < 0.05

An emerging number of studies suggest that EVs isolated from hypoxic cells can have functional consequences in several recipient cell types (Bister et al., 2020). One of such reported functions is the inhibition of pro‐inflammatory activation of immune cells. To test this in our study setting, we exposed the microglial N9 cell line to ultracentrifugation enriched EVs from the cobalt chloride treated N2A cells and followed the effects of the EVs on microglial responses to inflammation using proliferation and phagocytosis as functional read out measures. To mimic concomitant inflammation of the ischemic environment in vitro, bacterial LPS was used to induce pro‐inflammatory activation of microglia after the pre‐treatment with neuronal EVs isolated after different lengths of cobalt chloride treatment (Figure 6A,C). In the second in vitro design, microglia were exposed to different amounts of EVs without concomitant LPS (Figure 6B,D). We failed to show any significant differences in phagocytosis (Figure 6A,B) or proliferation (Figure 6C,D) rates of microglia after the pro‐longed repeated exposure to neuronal EVs isolated after 6‐, 12‐ or 24‐h cobalt chloride treatment compared to the exposure to vehicle treated EVs, or any dose response for neuronal EVs isolated from 24‐h cobalt chloride time point. Our results suggest that the EVs from hypoxic neurons do not modulate inflammatory responses in microglia in vitro in comparison to the EVs from control cells.

### 3.7 Peripheral blood levels of miR‐21‐5p correlate with the functional outcome in ischemic stroke patients

**FIGURE 7:**
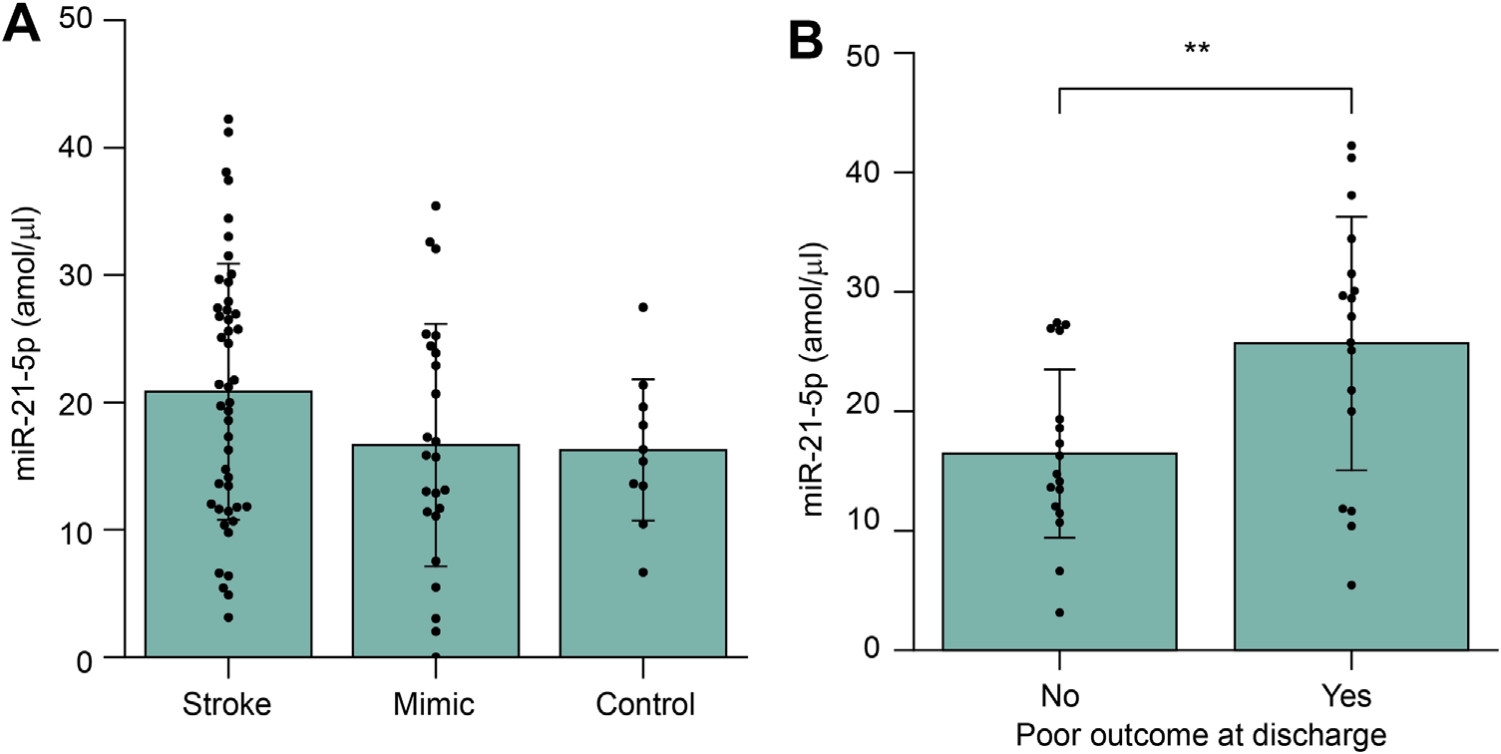
**Blood miR‐21‐5p level may predict the short‐term outcome of ischemic stroke**. **A**. miR‐21‐5p levels in ischemic stroke (n = 46), mimics (n = 23), and control subjects (n = 10). **B**. Acute miR‐21‐5p blood levels in patient with poor outcome at hospital discharge (n = 34). The data is represented as mean ± SD. The significance was determined by one‐way ANOVA with Tukey's post‐hoc (stroke vs. control *p* = 0.266 and stroke vs. control *p* = 0.201) for A and t‐test (*p* = 0.007) for B. Stated as ***p < 0.001, **p < 0.01, *p < 0.05

Finally, to evaluate whether the stroke induced increase of miR‐21a‐5p provides any biomarker potential in humans, we evaluated the levels of miR‐21‐5p, a human miRNA with completely identical mature sequence to the mouse miR‐21a‐5p, in the blood samples of ischemic stroke patients. Ischemic stroke patients showed a tendency to have increased levels of miR‐21‐5p (20.8 amol/ul) when compared to the stroke mimicking conditions (17.4 amol/ul) and control subjects (16.3 amol/ul) (Figure 7A).

Interestingly, 17 out of 34 the patients (50%) exhibited significant disability at hospital discharge (mRS 3 to 6). Ischemic stroke patients showing significant disability at discharge had significantly higher levels of miR‐21‐5p at admission (25.7 amol/ul) compared to those not showing significant disability (16.4 amol/ul) (Figure 7B). These data suggest that the acute level of peripheral blood miR‐21‐5p may predict the severity of ischemic stroke.

## 4 DISCUSSION

Neurons are one of the most sensitive brain cells to hypoxic insults. Recent interest in the involvement of EVs in cellular responses to hypoxia and cell‐to‐cell communication (reviewed in Bister et al., 2020) calls for closer evaluation of the methodological validity and applicability for studies involving EVs derived from sensitive CNS cells, as most of the existing studies focus on different tumour, cardiac and endothelial cells. Furthermore, altered release of several miRNAs in hypoxic EVs has been repeatedly reported. In particular, miR‐21a‐5p secretion has been reported to be increased in EVs under various conditions of hypoxia, especially in cancer (Guo et al., 2018; Li et al., 2019; Ren et al., 2019). When these EVs have been administered to other cells, they have been shown to mediate anti‐apoptotic and anti‐inflammatory functions, depending on the recipient cell type in question in mice models. In animal models of cerebral ischemia, miR‐21 has been shown to be induced in the brain tissues of transient MCAO in rats (Dharap & Vemuganti, 2010) and mice (Lee et al., 2010), and in an embolic MCAO in rats (Buller et al., 2010). These findings are in line with our data reported here for the permanent MCAO cerebral ischemia model in mice. Furthermore, increased neuronal level of miR‐21 has been previously reported in vivo after embolic ischemia (Buller et al., 2010) and in vitro after OGD (Ziu et al., 2011). Interestingly, a decreased miR‐21 level has been detected in the plasma from acute ischemic stroke patients (Zhou & Zhang, 2014) and in the peripheral blood from intracerebral hemorrhage patients (Wang et al., 2016). Post‐mortem human samples showed increased brain miR‐21 levels in subjects who had died from ischemic stroke compared to those who had died from drug abuse (Sessa et al., 2019). Finally, miR‐21 was shown to repress microglia‐mediated neuronal death (Zhang et al., 2012). All of these observations suggest the relevance of miR‐21a‐5p in response to brain ischemia and its possible release in EVs. However, studies on miR‐21 secretion in EVs in conditions of brain ischemia, either in vitro or in vivo, are missing. To our knowledge, the release of EVs from hypoxic neurons has been reported in only one study using a 6‐h hypoxia followed by a reperfusion period of 24 h (Wang et al., 2021). The reperfusion injury of neurons caused an altered EV‐associated release of miRNAs compared to normoxic controls and miR‐21‐5p was not reported in the study.

Here we show for the first time the time‐dependent release of EVs from hypoxic neurons without reperfusion. We studied the dynamics of hypoxia‐induced expression of miR‐21a‐5p in vivo and in vitro and suggest a role for miR‐21a‐5p in the regulation of pathways involved in vesicle homeostasis, inflammation and cell death. In addition, we show that miR‐21a‐5p is secreted outside the cells in the EVs and as non‐EV associated form. Interestingly, the increase in the levels of EV miR‐21a‐5p preceded the increase observed at the whole cell level, statistically significant increase being detectable in the EVs as early as two hours after the cobalt chloride exposure before any loss of cell viability was detectable. These cellular and vesicular kinetics of miR‐21a‐5p could partly depend on each other; while the EVs start to export miR‐21a‐5p out from the cells quickly upon hypoxic insult, a modest concomitant increase in miR‐21a‐5p production might not be detectable for some time. However, further studies are required to confirm this hypothesis as cellular miRNA metabolism is regulated at multiple different levels and, thus, the total amount of any mature miRNA in the cells is a result of miRNA synthesis, degradation, and release processes together (Kingston & Bartel, 2019). In addition to the changes observed in the vesicular level of miR‐21a‐5p, cryo‐EM imaging showed an abundance of larger EVs after 24 h hypoxia induced by cobalt chloride treatment, compared to any shorter exposures or vehicle treatments. From the cryo‐EM images, it is evident that hypoxia induced drastic changes in the formation of lipid bilayered vesicles, but the specific biogenetic origin of these vesicles remains to be discovered in detail.

Existing literature on the EVs does not often report how the utilised hypoxic insult influenced cell viability (Bister et al., 2020). Since multiple cell types are known to be sensitive to hypoxia, this makes it difficult to understand whether the observed effects were due to altered number of EVs, their altered cargo or side effects caused by cell death itself. Apoptosis is a programmed cell death mechanism that does not cause uncontrolled rupturing of the plasma membrane or the release of intracellular contents, opposite to what occurs during necrotic cell death. To date, apoptotic mechanisms have been thoroughly characterised for the formation of relatively large apoptotic bodies (Wickman et al., 2013) but detailed studies analysing the formation of smaller, nanosised, particles remain a less studied topic. Some early investigations of these smaller vesicles, often called apoptotic EVs or apoEVs, do exist (Théry et al., 2001; Tucher et al., 2018) and the data obtained in our study call for the importance of studying these apoptosis‐induced small vesicles in detail. Indeed, apoEVs from UVB irradiated T cells have leaky membranes, being an opposite of what has been described for the EVs released by healthy cells in which the EV membrane is considered to protect the cargo (Schiller et al., 2012). In addition, several histones are exclusively released in EVs by apoptotic dendritic cells, not from healthy cells (Théry et al., 2001). Future studies should focus on how, if necessary, to distinguish the apoptosis‐induced small EVs from the EVs released by healthy cells.

We fractionated the conditioned medium from neurons using SEC to separate the EVs and the bulk of proteins to analyse in detail in which form miR‐21a‐5p was secreted outside the cells. Our data show that the miR‐21a‐5p is much more abundant in the bulk protein fraction as a non‐EV associated form, compared to EV fraction. This observation is in line with others showing that only a few percent of total extracellularly secreted miRNA from in vitro cell culture models is EV associated (Sork et al., 2021). Similarly, miR‐124‐3p, miR‐23a and miR‐122 have been previously detected at higher levels in the protein fractions than the EV fractions separated from plasma using SEC (Karttunen et al., 2019). However, we additionally show that the small fraction of total miR‐21a‐5p that was secreted inside EVs is well protected from enzymatic degradation. This observation could have biological and practical relevance, supporting the stability of the EV loaded miR‐21a‐5p and could be taken an advantage of in the EV‐based biomarker discovery.

Despite several studies showing different responses of phagocytes to EVs enriched from hypoxic MSCs (Cui et al., 2018; Li et al., 2015; Ren et al., 2019) and oncogenic cells (Chen et al., 2018; Guo et al., 2018; Li et al., 2019), we were not able to detect any functional effects of hypoxic neuron derived EVs in the microglial cells. The tested responses included proliferation and phagocytosis. Importantly, to our knowledge this is the first study where specifically hypoxic neuron derived EVs were administered to microglia, with or without concurrent inflammatory stimulus by LPS. It is possible that the neurons do not functionally communicate with microglia through the direct transfer of EVs under hypoxia, which could explain the lack of functional modulation in our in vitro design. There are also several other plausible explanations, both biological and technical, for the lack of significant responses in our study. The EVs generated by hypoxic neuronal cells could be taken up by microglia differently than the EVs generated in normoxic conditions. Also, the faith of the EVs and EV cargo in microglia can differ between the hypoxic and normoxic EVs. For example, certain conditions might promote degradation of the EVs in the lysosomes compared to other faiths, a phenomenon that is not well studied. Furthermore, hypoxic neuron derived EVs might carry several molecules having opposite effects in microglia, diluting the response frequently reported for the EV delivered miR‐21a‐5p in myeloid cells (Xin et al., 2020). This might have been especially true upon prolonged hypoxic exposure of the EV producing cells. However, with shorter exposures the EV yield is limited as the EV secretion during the first hours was very modest and often required pooling of samples and regardless, the measurements were often carried out close to the lower detection limits. This could have introduced variance to the study design. Further studies are required to increase the confidence on the hypoxic neuron communication with microglia via EVs, focusing on the detailed characterisation of the EVs, using primary or stem cell derived cell models. Additional future directions would be to also evaluate the effects of hypoxic neuronal EVs in other relevant cell types, such as endothelial cells or astrocytes, affected by inflammation.

From a clinical biomarker point of view, three studies demonstrate an increase in the serum level of miR‐21‐5p in patients with ischemic stroke (Tsai et al., 2013; Wu et al., 2017; Xiang et al., 2017). Tsai et al. detected increased miR‐21a‐5p levels in ischemic stroke patient serum samples obtained within 7 days after hospital admission. Wu et al. showed that miR‐21a‐5p was associated with higher severity of ischemic stroke but failed to predict increased stroke risk following transient ischemic attack or the clinical outcome of the patients. Xiang et al. showed similar correlation of increased miR‐21‐5p with stroke but could not find association between miRNA polymorphism and miR‐21‐5p expression. Thus, whilst the ability of ischemic stroke to increase the serum levels of miR‐21‐5p is well established, our study using blood is the first to suggest that peripheral miR‐21‐5p may have value in the prediction of the clinical outcome of the patients. Indeed, we demonstrate that increased levels of blood miR‐21‐5p at admission predict worsened outcome of the patients at hospital discharge. Moreover, our study is the first to obtain the quantification of miR‐21‐5p in human samples with an immunoassay instead of qPCR, likely providing better accuracy and producibility. In addition, we are the first ones to report the association of miR‐21‐5p with stroke outcome in a Caucasian population.

To summarise, here we pinpoint miR‐21a‐5p as the most prominently deregulated neuronal miRNA in a mouse model of ischemic stroke. miR‐21a‐5p is packed in EVs even prior to the hypoxia‐induced increase of miR‐21a‐5p at the cellular level. At the same time, we show that the majority of miR‐21a‐5p is secreted in non‐EV associated form. Whilst the impact of the EVs failed to alter microglial functions, the secreted miR‐21‐5p may provide biomarker potential for predicting significant disability at hospital discharge. This may have clinical value by indicating which set of patients may require more extensive care after the incidence of stroke.

## AUTHOR CONTRIBUTIONS

**Nea Korvenlaita**: Conceptualization; Data curation; Formal analysis; Funding acquisition; Investigation; Methodology; Visualization; Writing – original draft. **Mireia Gómez‐Budia**: Methodology; Visualization; Writing – original draft. **Flavia Scoyni**: Investigation; Methodology. **Cristiana Pistono**: Methodology. **Luca Giudice**: Formal analysis. **Shaila Eamen**: Methodology. **Sanna Loppi**: Methodology. **Benjamin Huremagic**: Formal analysis. **Maria Bouvy‐Liivrand**: Formal analysis. **Merja Heinäniemi**: Formal analysis; Supervision. **Lesley Cheng**: Formal analysis; Methodology. **Laura Ramiro**: Methodology. **Joan Montaner**: Investigation; Resources. **Tereza Batkova**: Methodology. **Robert Mikulik**: Investigation; Resources. **Rosalba Giugno**: Formal analysis; Supervision. **Jukka Jolkkonen**: Writing – original draft; Writing – review & editing. **Paula Korhonen**: Methodology; Writing – review & editing. **Tarja Malm**: Conceptualization; Funding acquisition; Project administration; Resources; Supervision; Writing – review & editing

## CONFLICT OF INTEREST

Authors report no conflicts of interest.

## GEOLOCATION INFORMATION

Kuopio, Finland.

## Supporting information

Supplementary Information

Supplementary Figure 1. N2A cell viability in serum‐free conditions and following GW4869 treatment.
Supplementary Figure 2. The level of miR‐127‐5p in EVs isolated from hypoxic N2A cells.
Supplementary Figure 3. Characterization of extracellular vesicle and protein fractions after Size Exclusion Chromatography.
Supplementary file 1. Gene targets of miRNAs from HITS‐Clip sequencing analysis and enriched pathways of selected miRNAs.

Supplementary Information

Supplementary Information

Supplementary Information

## Data Availability

The data discussed in this publication have been deposited in NCBI's Gene Expression Omnibus and are accessible through GEO Series accession numbers GSE215210 (GRO‐sequencing), GSE215211 (HITS‐CLIP sequencing) and GSE215212 (small RNA sequencing).
