# Supplementary material for: Dynamic release of neuronal extracellular vesicles containing miR‐21a‐5p is induced by hypoxia
Source: J Extracell Vesicles. 2023 Jan 3;12(1):12297. doi: 10.1002/jev2.12297 (PMC9809533; doi:10.1002/jev2.12297)
Supplement: Supplementary file 2 — Supplementary Figure 1. N2A cell viability in serum‐free conditions and following GW4869 treatment. Supplementary Figure 2. The level of miR‐127‐5p in EVs isolated from hypoxic N2A cells. Supplementary Figure 3. Characterization of extracellular vesicle and protein fractions after Size Exclusion Chromatography. Supplementary file 1. Gene targets of miRNAs from HITS‐Clip sequencing analysis and enriched pathways of selected miRNAs. [file JEV2-12-12297-s003.docx]

# Supplementary file captions

**Supplementary Figure 1.** **N2A cell viability in serum-free conditions and following GW4869 treatment.** **A.** Viability of N2A cells measured by MTT assay. **B.** Viability of N2A cells measured by flow cytometry using DAPI staining at 24 hours. **C.** Viability of N2A cells measured by MTT assay at 24 hours.

**Supplementary Figure 2.** **The level of miR-127-5p in EVs isolated from hypoxic N2A cells.** The levels of miR-127-5p measured by qPCR in ultracentrifugation purified N2A EVs 6 hours after (**A**) cobalt chloride or (**B**) oxygen-glucose deprivation treatment.

**Supplementary Figure 3. Characterization of extracellular vesicle and protein fractions after Size Exclusion Chromatography.** **A.** Bar graphs show relative purity of EVs in different fractions based on NTA measured particle number (right y-axis) and line indicates total protein concentration measured by BCA (left y-axis). Grey areas indicate enriched EV and protein fractions. **B.** Size distributions of SEC purified EV and protein fractions measured by NTA. Length of vehicle and cobalt chloride treatment of N2A cells is 24 hours.

**Supplementary file 1. Gene targets of miRNAs from HITS-Clip sequencing analysis and enriched pathways of selected miRNAs.**
