# Supplementary figures and images for: Dynamic release of neuronal extracellular vesicles containing miR‐21a‐5p is induced by hypoxia
Source: J Extracell Vesicles. 2023 Jan 3;12(1):12297. doi: 10.1002/jev2.12297 (PMC9809533; doi:10.1002/jev2.12297)

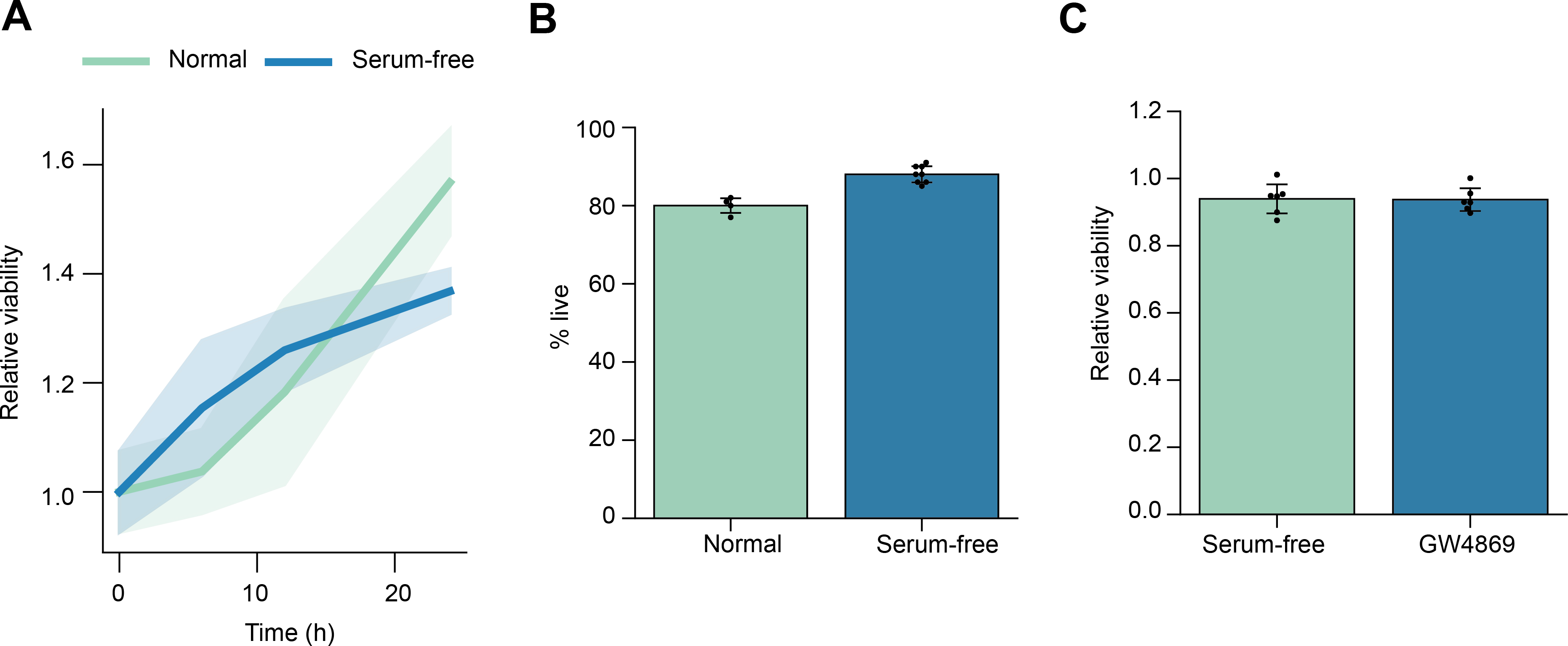

Supplement: Supplementary file 3 — Supplementary Information [file JEV2-12-12297-s002.png]

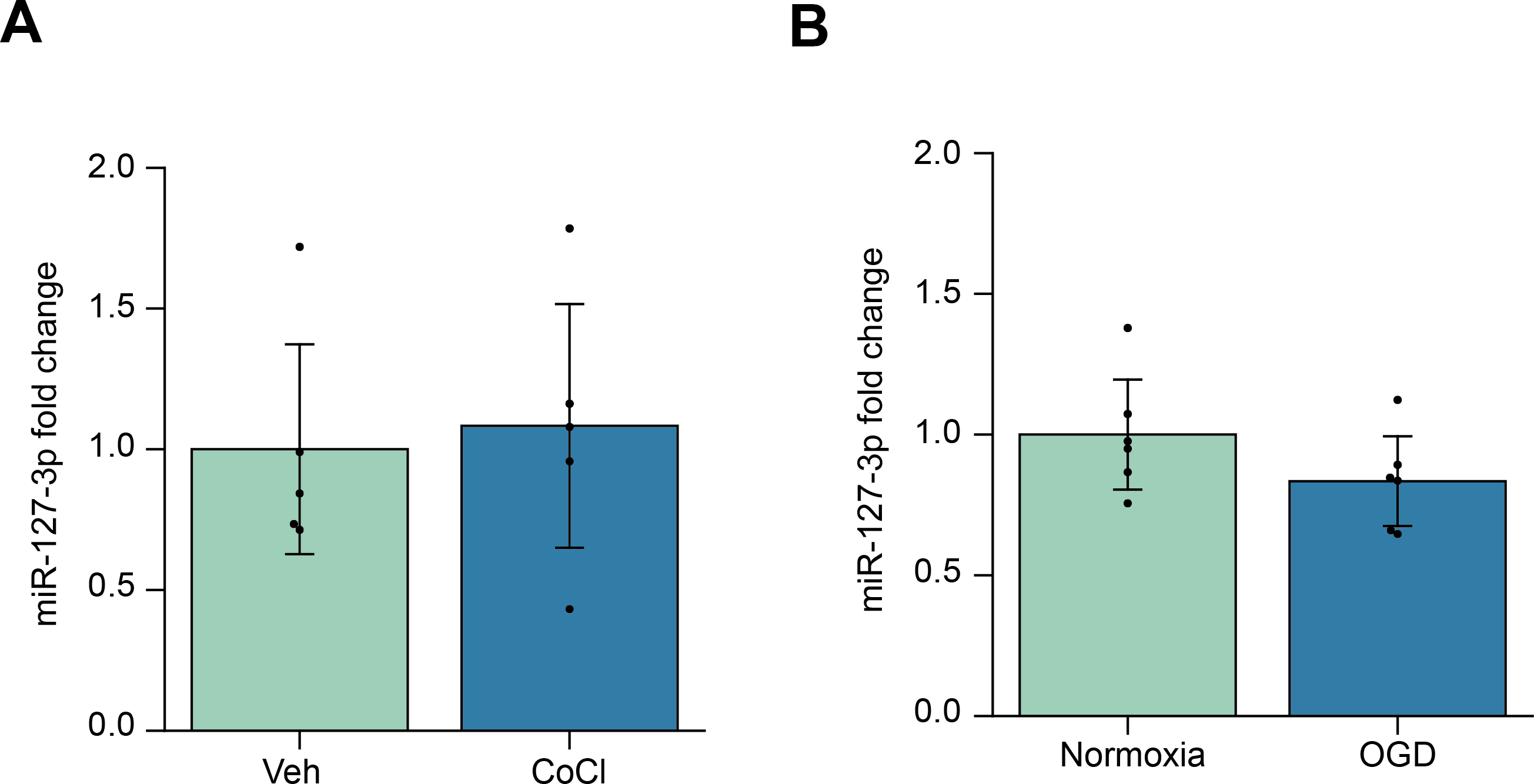

Supplement: Supplementary file 4 — Supplementary Information [file JEV2-12-12297-s001.png]

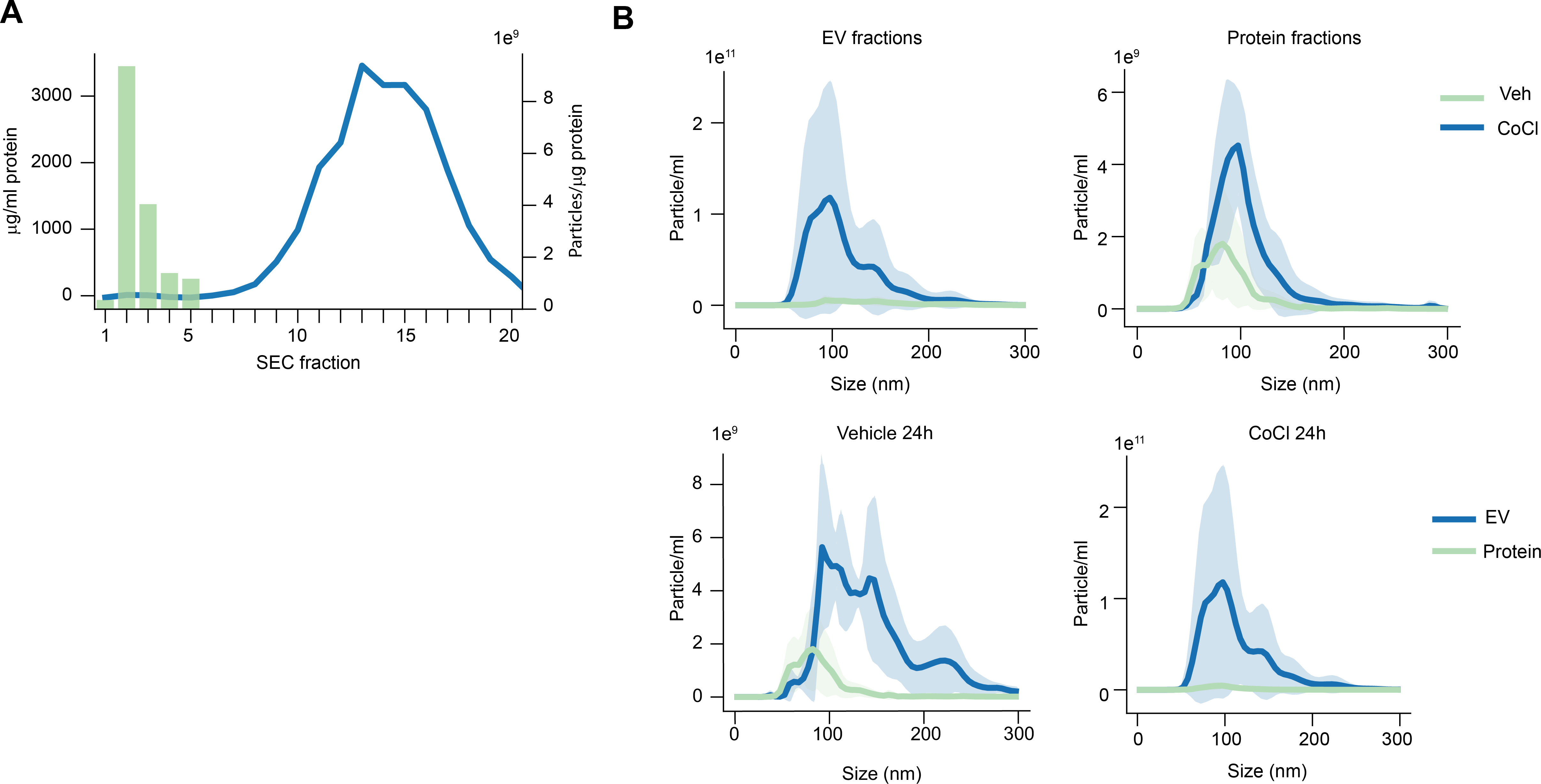

Supplement: Supplementary file 5 — Supplementary Information [file JEV2-12-12297-s005.png]
